# Three-Dimensional Human Liver Micro Organoids and Bone Co-Culture Mimics Alcohol-Induced BMP Dysregulation and Bone Remodeling Defects

**DOI:** 10.3390/cells15030274

**Published:** 2026-02-01

**Authors:** Yuxuan Xin, Guanqiao Chen, Mohammad Majd Hammour, Xiang Gao, Fabian Springer, Elke Maurer, Andreas K. Nüssler, Romina H. Aspera-Werz

**Affiliations:** 1 Department of Traumatology, Siegfried Weller Institute, BG-Klinik Tübingen, Eberhard Karls University, 72076 Tübingen, Germany; yuxuanxin.orthopedics@gmail.com (Y.X.); guanqiaochen0@gmail.com (G.C.); m.hammour@hotmail.com (M.M.H.); xianggao23@gmail.com (X.G.); romina.aspera-werz@med.uni-tuebingen.de (R.H.A.-W.); 2 Department of Orthopaedic Surgery, The First Affiliated Hospital of Shandong First Medical University & Shandong Provincial Qianfoshan Hospital, Jinan 250014, China; 3 Department of Diagnostic and Interventional Radiology, University Hospital Tübingen, Hoppe-Seyler-Str. 3, 72076 Tübingen, Germany; fabian.springer@med.uni-tuebingen.de; 4 Department of Radiology, BG-Klinik Tübingen, Eberhard Karls University, 72076 Tübingen, Germany; 5 Department Traumatology and Reconstructive Surgery BG-Clinic Tübingen, 72076 Tübingen, Germany; 6 Lumedis Orthopedics, Kirchstr. 12, 60311 Frankfurt, Germany

**Keywords:** hepatic osteodystrophy, liver micro-organoids, liver-bone co-culture system, alcohol, alcohol-induced bone loss, liver-bone axis, BMP signaling

## Abstract

**Highlights:**

**What are the main findings?**

- The present study established a long-term 3D human liver–bone co-culture model mimicking alcohol-induced hepatic osteodystrophy (HOD) with fibrogenic liver changes and bone defects.
- Chronic 50 mM alcohol triggers hepatic CYP2E1 activation, EMT/fibrosis, BMP imbalance (↓BMP2, ↑BMP13), reduced osteoblast mineralization, and chondrogenic shift in bone progenitors.

**What are the implications of the main findings?**

- The present study provides an organoid-based platform for studying the liver–bone axis and BMP dysregulation in HOD, surpassing monocultures or animal models.
- The present study enables the screening of BMP-targeted therapies to restore bone homeostasis in alcohol-related chronic liver disease.

**Abstract:**

Hepatic osteodystrophy (HOD) is a frequent complication of chronic liver disease, marked by impaired osteogenesis and elevated fracture risk, particularly under sustained alcohol exposure. Bone morphogenetic proteins (BMPs), which play a crucial role in maintaining bone homeostasis, are dysregulated in alcoholic liver disease. Specifically, decreased BMP2 and increased BMP13 have been linked to impaired osteogenesis and cartilage-like shifts in bone progenitors. A human in vitro system that recapitulates this hepatic BMP imbalance is needed to dissect mechanisms and identify targets. To address this, we established a long-term human three-dimensional liver–bone co-culture model that integrates hepatocytes (HepaRG), hepatic stellate cells (LX-2), and human umbilical vein endothelial cells (HUVECs) with bone scaffolds seeded with osteoblast precursors (SCP-1) and osteoclast precursors (THP-1). This study aimed to characterize the effects of chronic 50 mM alcohol exposure on hepatic fibrogenic activation and BMP ligand secretion, and to investigate the associated BMP-responsive signaling involved in bone cell lineage differentiation and functional activity. The results demonstrated alcohol-induced hepatic CYP2E1 activation and fibrogenic remodeling with EMT signatures, as well as a decrease in BMP2 and an increase in BMP13, without affecting BMP9. Liver-derived factors activated both canonical and non-canonical BMP signaling in bone progenitors, reduced osteoblast activity and mineralization, preserved osteoclast TRAP activity, and shifted the lineage toward chondrogenesis (*SOX9*↑, *RUNX2*↓). Notably, this BMP profile and skeletal phenotype reflect clinical observations in chronic liver disease, indicating that the model recapitulates key in vivo pathological features. This human liver micro-organoid co-culture reproduces alcohol-induced hepatic BMP dysregulation and downstream bone defects, offering an organoid-centric, microengineered platform for mechanistic studies and BMP-targeted therapeutic screening in HOD.

## 1. Introduction

Hepatic osteodystrophy (HOD) is a common complication secondary to chronic liver disease (CLD), mainly characterized by decreased bone mineral density, altered bone microarchitecture, and increased fracture risk [1,2]. This bone metabolic disorder significantly compromises patients’ quality of life and long-term prognosis. Although its underlying pathogenesis remains incompletely understood, growing evidence suggests that liver dysfunction may promote bone loss and disrupt bone homeostasis through systemic metabolic disturbances and dysregulation of liver–bone axis signaling [3,4]. Among the various etiologies of CLD, chronic alcohol abuse represents one of the most prevalent and well-characterized causes [5,6]. According to the World Health Organization, approximately 43% of individuals aged 15 years and older consume alcohol [7], with an average annual pure alcohol intake of 6.2 L per person [8]. In this study, we aim to investigate how liver injury, modeled through chronic alcohol exposure, leads to dysregulation of bone metabolism via impaired liver–bone crosstalk.

Existing studies indicate that chronic alcohol exposure is a major driver of hepatic fibrosis, characterized by excessive extracellular matrix deposition and activation of hepatic stellate cells (HSCs) [9,10]. Emerging evidence suggests that bone morphogenetic proteins (BMPs) play critical roles in this process. In the fibrotic liver, BMP2 expression is often downregulated, impairing hepatocyte regeneration and favoring a profibrotic environment [11]. BMP9, which normally contributes to vascular stability and hepatocellular homeostasis, exhibits dysregulated expression under alcoholic stress, thereby exacerbating endothelial dysfunction and fibrotic remodeling [12]. Conversely, BMP13 is markedly upregulated during hepatic fibrogenesis, where it promotes HSC activation and collagen synthesis [13]. Such fibrosis-associated alterations in hepatic BMP expression may not only accelerate liver injury but also exert systemic effects on distant organs, particularly bone tissue. Indeed, liver injury has been shown to disrupt bone remodeling through both direct and indirect mechanisms. Direct effects may involve the toxicity of alcohol or liver-derived metabolites on osteoblasts and osteoclasts, whereas systemic metabolic disturbances, hormonal imbalances, and chronic inflammation mediate indirect effects. Given that BMPs are key regulators of osteoblast differentiation and bone homeostasis, hepatic BMP dysregulation may represent a crucial molecular link between chronic liver injury and skeletal disorders. In the bone microenvironment, BMP2 is essential for osteoblast differentiation and mineralization [14], while BMP9 regulates vascular integrity and bone metabolism [15]. By contrast, BMP13 is associated with fibrogenic and chondrogenic pathways [16]. Clinical studies have shown that patients with long-term liver disease exhibit decreased circulating BMP2 and increased BMP13 levels, correlating with reduced bone mineral density and cartilage-like changes in the bone marrow [11,13]. Despite these findings, the specific role of liver-derived BMPs in alcohol-induced bone homeostatic imbalance remains unclear, partly due to the lack of physiologically relevant in vitro models capable of recapitulating long-term liver–bone interactions. Therefore, a human in vitro system that accurately reproduces this hepatic BMP imbalance is needed to dissect the underlying mechanisms and identify potential therapeutic targets.

While animal models have provided valuable insights into alcohol-induced liver and bone pathology, their translational relevance to HOD remains limited due to interspecies differences and ethical constraints [3,17]. Although BMPs share central functions between humans and animal models, there are important differences in the magnitude of response, receptor expression, and specific functional outcomes [18]. Although human in vitro liver–bone co-culture systems have been developed previously, they often lack essential fibrogenic components, such as HSCs, and therefore fail to capture the key pathological features of liver injury [19]. Consequently, the establishment of long-term three-dimensional (3D) co-culture systems that sustain physiological liver–bone interactions and mimic fibrotic progression is urgently required.

In Europe and North America, alcohol-related liver disease represents a leading cause of chronic liver disease and cirrhosis, accounting for nearly half of all cases [20,21,22]. Although the hepatotoxic mechanisms of alcohol have been extensively characterized, the consequences of liver dysfunction on skeletal homeostasis via the liver–bone axis remain poorly understood. Critically, long-term human in vitro systems modeling hepatic BMP dysregulation and its skeletal sequelae are lacking—a pivotal knowledge gap that our study addresses through innovative 3D co-culture models. Our findings elucidate these mechanisms, offering novel insights into hepatic osteodystrophy pathogenesis.

## 2. Materials and Methods

### 2.1. Cell Culture

Details of the culture media for all cells are provided in Supplementary Table S1.

HepaRG cells (Biopredic International, Saint Grégoire, France), which are liver progenitor cells able to differentiate into hepatocytes and biliary cells, were maintained in the undifferentiated state in HepaRG Culture medium at 37 °C and 5% CO_2_. During the proliferation phase, HepaRG cells were passaged every 2 weeks. Cell differentiation was induced using 1.7% dimethyl sulfoxide (DMSO; 4720.2, Roth) for another 2 weeks [23]. During HepaRG proliferation and differentiation, medium changes were performed every other day.

The human hepatic stellate cell line, LX-2 (Merck Millipore, Darmstadt, Germany), was cultured in LX-2 culture medium. Cells were maintained in a humidified incubator at 37 °C with 5% CO_2_ [24]. All experiments were conducted using LX-2 cells at passages 5 to 14, with the culture medium refreshed twice weekly.

HUVECs [25] (kindly provided by Prof. Alexander-Friedrich) belonging to the umbilical vein endothelial cell line were cultured in HUVEC culture medium on the 0.1% gelatine-coated flask. All the experiments with HUVECs were performed at passages 7 to 12. The medium was changed twice weekly.

For the human leukemia monocytic cell line, THP-1 (ACC16, DSMZ) cells, as the osteoclastic precursor cells, were cultured as a suspension cell culture in THP-1 culture medium [19]. The cells were maintained in a humidified atmosphere with 5% CO_2_ at 37 °C, and the growing medium was renewed twice weekly.

SCP-1, a mesenchymal stem cell line [26], (kindly provided by Prof. Dr. Matthias Schieker) was used as osteoprogenitor cells and cultured in SCP-1 culture medium at 37 °C in a humidified atmosphere with 5% CO_2_. The growing medium was refreshed twice weekly.

### 2.2. Chemical and Reagents

All chemicals were purchased from Sigma-Aldrich (St. Louis, MO, USA) or Carl Roth (Karlsruhe, Germany).

### 2.3. Preparation and Sterilization of Agarose Plates

Non-adherent agarose microwell plates were fabricated using mold replication technology, as mentioned previously [27]. Briefly, molten agarose solution (2%, 3.2 mL; Lonza, Basel, Switzerland, 50004) was dispensed into individual wells of a 6-well plate. A polydimethylsiloxane insert (generously provided by Prof. Massoud Vosough), featuring 300 pyramid-shaped microwells with a diameter of 800 µm, was then placed onto the surface. Once the agarose solidified, the insert was removed, creating the desired microwell structure [28]. The plates could be stored at 4 °C for up to 2 weeks if sealed to prevent drying. Before use, the plates were sterilized under ultraviolet light for 1 h.

### 2.4. Preparation of Human Platelet-Rich Plasma (hPRP) Scaffolds

PRP scaffolds were generated as reported previously [29]. The thoroughly mixed solution was kept on ice and contained 16.0% 2-Hydroxyethyl methacrylate (128635-500G, Sigma), 0.3% Bis-Acrylamide (3039.1, Carl Roth), 0.25 g/L PRP, and ddH_2_O. After 30 min of incubation, di-sodium hydrogen phosphate buffer (T876.1, Carl Roth) was added to the mixture to reach a final concentration of 0.3 M. Subsequently, 0.1% glutaraldehyde (3778.1, Carl Roth), 0.2% Ammonium Persulfate (A3678-25G, Sigma), and 0.2% TEMED (2367.3, Carl Roth) were incorporated into the reaction mixture. The final mixture was transferred to the 6 mm diameter, 3 mm height polystyrene molds (2 mL per mold), then frozen at −20 °C overnight. To facilitate cutting, the molds were transferred at −80 °C for at least half an hour before operation. The resulting scaffolds exhibited uniformity in size, with a height of 4 mm and a diameter of 6 mm. The HEMA-based scaffolds were then transferred to a 1 M CaCl_2_ (CN93.2, Carl Roth) solution and placed on a rotating shaker overnight. Then the scaffolds were transferred into 70% alcohol and incubated overnight on a rotating shaker for at least 12 h. Finally, the scaffolds were washed with sterile Phosphate-Buffered Saline (PBS) 4 times and stored at 4 °C with sterile PBS. Before use, the fabricated scaffolds were placed in a 48-well plate and incubated in THP-1 culture medium for 48 h to verify sterility and ensure proper preconditioning.

### 2.5. Cell Seeding

#### 2.5.1. Bone Co-Culture System

For the 3D bone co-culture system, THP-1 and SCP-1 cells were seeded onto the PRP scaffold. Following the method previously described [30], THP-1 cells (8 × 10^4^ cells per scaffold, suspended in 15 µL) were applied to the scaffold surface in THP-1 culture medium supplemented with 200 nM phorbol 12-myristate 13-acetate (PMA; Cay10008041, Biomol, Hamburg, Germany). Following a 4 h incubation at 37 °C under humidified conditions with 5% CO_2_, an additional 500 µL of THP-1 culture medium containing 200 nM PMA was added to each scaffold. The following day, the cell culture medium was removed, and SCP-1 cells (1 × 10^4^ cells/15 µL per scaffold) were seeded in bone co-culture differentiation medium. SCP-1 cells were also incubated for 4 h to allow them to adhere, after which, 500 µL of bone co-culture differentiation medium was added to each scaffold [31]. The bone co-culture scaffolds are maintained in a humidified 37 °C environment with 5% CO_2_, with the medium changed twice weekly.

#### 2.5.2. Liver Micro-Organoids

For the 3D liver micro-organoid model, differentiated HepaRG cells, LX-2 cells, and HUVECs were seeded at a ratio of 4:2:1 in pre-prepared agarose microwells within a 6-well plate [28]. To begin, each well was filled with 2 mL of liver micro-organoid culture medium. Then, 1 mL of cell suspension, prepared at a density of 3.0 × 10^5^ cells/mL (1000 cells per microwell) in the same medium, was added to each well. To ensure an even distribution of cells within the microwells, the plate was immediately centrifuged for 3 min at 1200 rpm at room temperature. The spheroids were cultured in a humidified incubator at 37 °C with 5% CO_2_. The liver micro-organoid culture medium was refreshed three times per week.

#### 2.5.3. Development of 3D Human In Vitro Liver–Bone Co-Culture System

**Figure 1:**
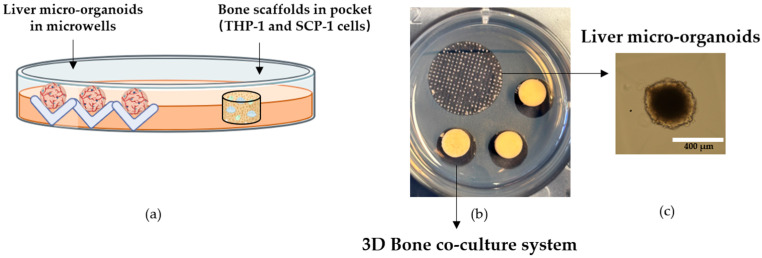
The structure of the 3D human in vitro liver–bone co-culture system. (**a**) Schematic illustration showing the setup of liver micro-organoids formed in microwells and bone scaffolds (containing THP-1 and SCP-1 cells) placed in agarose pockets. (**b**) Photograph of the 3D liver–bone co-culture system. (**c**) Representative microscopy image of a liver micro-organoid (white scale bar = 400 μm).

When liver micro-organoids had formed, the side of the agarose without cell distribution was hollowed out to create a pocket shape for placing the scaffold. The ratio of liver micro-organoids to bone scaffolds was 300:3 (Figure 1). After combining to form the liver–bone system, the co-culture system was maintained using liver–bone co-culture medium for 28 days [19]. The medium is changed three times per week. The liver–bone co-culture system is cultured in a humidified incubator, maintaining a temperature of 37 °C and 5% CO_2_.

#### 2.5.4. Stimulation of the Liver–Bone System with Alcohol

Absolute alcohol (20821.330, VWR, Radnor, PA, USA) was stored at 4 °C. To prepare alcohol-containing medium, 3, 6, or 12 μL of alcohol was added to 1 mL of complete medium to achieve final concentrations of 50, 100, and 200 mM, respectively. The co-cultured system was stimulated with alcohol daily, and the complete medium was refreshed twice a week. To minimize alcohol evaporation, the culture plates were covered with sealing membranes (391-1262, VWR, Radnor, USA) during incubation.

### 2.6. Measurement of Mitochondrial Activity

The Resazurin conversion assay was used to measure the metabolic activity of the cells [19]. For the liver micro-organoids, the spheroids were collected from agarose and washed with PBS prior to analysis. Then, 300 µL of 0.0025% resazurin working solution was used for a total of 300 liver micro-organoids, as the background 100 µL resazurin working solution without incubation with cells was used. From this, three 100 µL aliquots of liver micro-organoids in the resazurin working solution were transferred to a 96-well plate. After incubation for 30, 60, 90, and 120 min at 37 °C, the plate reader FLUOstar Omega Plate Reader V. 5.70 (BMG Labtech, Ortenberg, Germany) was used to measure the fluorescence produced by the resorufin at ex/em 544 nm/590–10 nm. For the bone portion, scaffolds were first washed with PBS, followed by the immediate addition of 500 µL of a 0.0025% resazurin solution. Scaffolds without cells served as background controls. After a 2 h incubation at 37 °C, a 96-well plate was filled with 4 × 100 µL of each scaffold sample to allow for the evaluation of the fluorescence by a similar detection method as liver micro-organoids.

### 2.7. Measurement of UDP-Glucuronosyltransferase (UGT) Activity (Phase II Enzymes)

To assess UGT activity, a fluorescence-based assay using 4-methylumbelliferone (4-MU) was performed. A total of 200 μL of 6.25 μM 4-MU, prepared in HepaRG plain medium, was applied to 300 liver micro-organoids. From this mixture, 100 μL was transferred into the wells of a 96-well plate containing the liver micro-organoids to generate duplicate samples. Additionally, 100 μL of the same working solution was added to a well without cells to serve as a background control [32]. The plate was incubated, and fluorescence was measured at excitation/emission wavelengths of 355 nm/460 nm using an Omega FLUOstar Plate Reader V. 5.70 (BMG Labtech, Ortenberg, Germany) at the following time points: 30, 60, 90, and 120 min. DNA concentration from the liver micro-organoids was quantified and used as a normalization factor.

### 2.8. Measurement of Alkaline Phosphatase (AP) Activity

AP activity detection was used to evaluate osteogenic differentiation and bone-forming cell function [33]. Scaffolds, washed with PBS, were incubated in 500 µL of AP reaction buffer (1 mM MgCl_2_, 50 mM glycine, 3.5 mM pNPP, and 100 mM Tris buffer; pH 10.5) for 2 h at 37 °C. The conversion of 4-nitrophenyl-phosphate to 4-nitrophenol was quantified photometrically (λ = 405 nm; Omega FLUOstar Plate Reader V. 5.70, BMG Labtech, Ortenberg, Germany) in quadruplicate from 100 µL samples of the AP reaction buffer. The experimental values obtained were adjusted using a background control (consisting of cell-free scaffolds in AP reaction buffer). The data were normalized using the data from the total DNA content of the bone co-culture system [34].

### 2.9. Measurement of Tartrate-Resistant Acid Phosphatase (TRAP) Activity

To evaluate osteoclast function, the TRAP level (a late-stage marker of osteoclast activity) was quantified [35]. In each assay, 30 µL of supernatant was combined with 90 µL of TRAP activity assay solution (containing 5 mM pNPP, 50 mM disodium tartrate at pH 5.5, and 100 mM sodium acetate) and incubated at 37 °C for 6 h. Subsequently, 90 µL of 1 M NaOH per well was added to inhibit further enzymatic activity. The reaction product, 4-nitrophenol, was quantified at 405 nm using a photometer. The experimental values were adjusted by subtracting the background control, which consisted of scaffolds without cells in the TRAP activity assay solution, and the data were normalized to the DNA content of the bone co-culture system [34].

### 2.10. DNA Isolation and Quantification

DNA measurements were performed to assess cell number and to normalize enzymatic activity values. For the liver micro-organoids, samples were first collected from the agarose microplate, washed with PBS, and then treated with 100 µL of 98 °C 50 mM NaOH. After vortexing, samples were stored at −20 °C for at least 24 h. The next day, they were thawed for 30 min (98 °C) and mixed with 110 μL of 0.1 M Tris (pH 8.0), and 100 µL of the mixture was transferred into a 96-well plate (V-type) and centrifuged at 20,000× *g* for 10 min. For the bone portion, bone scaffolds were washed with PBS, and 250 µL of 98 °C 50 mM NaOH was added to each scaffold. After 5 min of incubation, the scaffolds were frozen at −20 °C for at least 24 h. The next day, 275 μL of 0.1 M Tris (pH 8.0) was added to the thawed samples. The mixture was mixed thoroughly, transferred to a 96-well plate (V-type), and centrifuged at 20,000× *g* for 10 min. To determine the DNA concentration, absorption was measured at λ = 260 nm for all samples using a BMG Labtech LVIS, and the data were processed with Omega analysis software MARS software 3.42 [19].

### 2.11. Intracellular Lactate Dehydrogenase (LDH) Activity

LDH release into the culture medium is a widely used indicator of cell membrane damage [36]. However, in our in vitro liver–bone co-culture system, it is not feasible to distinguish the cellular origin of LDH detected in the supernatant, since both liver micro-organoids and bone-derived cells contribute to the extracellular LDH pool. To specifically assess intracellular LDH activity, assessment via CytoTox-ONE (Promega, Madison WI, USA) was performed in each tissue type. Liver micro-organoids and the bone system were collected separately and subjected individually to lysis with 1% Triton X-100 to fully release intracellular LDH. Subsequently, 50 μL of each lysate was mixed with 50 μL of LDH reaction working solution, and absorbance was measured at 490 nm using a FLUOstar Omega plate reader V. 5.70 (BMG Labtech, Ortenberg, Germany). The background absorbance, measured from wells without cells containing the LDH reaction working solution and Triton X-100, was subtracted from all sample values.

### 2.12. Luciferase Reporter Assay

To evaluate the nuclear activation of the BMP signaling pathway, a luciferase reporter assay was conducted using an adenoviral vector carrying a luciferase reporter gene under the control of a BMP-responsive element (Ad-BRE). SCP-1 cells were seeded into 96-well plates and infected with Ad-BRE for 24 h. After infection, cells were washed with PBS, then treated with the supernatant from liver micro-organoids stimulated by 50 mM alcohol. Following 48 h of stimulation, lysis buffer was added to each well to lyse the cells. The plates were subsequently stored at −80 °C for at least 1 h. Then, 30 μL of cell lysate was collected from each well and mixed with an equal volume (30 μL) of luciferase assay reagent. Luciferase activity was performed according to the manufacturer’s instructions, using the Steady-Glo Luciferase Assay System (Promega, Madison, WI, USA), and normalized to total protein content, measured with Lowry [37].

### 2.13. Semi-Quantitative Reverse-Transcription Polymerase Chain Reaction (RT-PCR) Analysis

Gene expression levels were determined by RT-PCR [28]. Total RNA was isolated from liver micro-organoids and bone system samples using TriFast reagent (Peqlab, Erlangen, Germany), and RNA concentration was determined with an Omega FLUOstar Plate Reader V. 5.70 (BMG Labtech, Ortenberg, Germany). Complementary DNA (cDNA) was generated using a commercial reverse transcription kit (Thermo Fisher Scientific, Waltham, MA, USA). Then, PCR amplification was performed according to the manufacturer’s instructions for Biozym Red HS Taq Master Mix (Vienna, Austria). Primer sequences and PCR conditions for target genes are shown in Supplementary Table S2; 18S rRNA served as a housekeeping gene. PCR products were resolved on 1.8% agarose gels containing ethidium bromide and separated by electrophoresis at 90 V for 60 min. Band intensities were subsequently quantified using ImageJ software V. 1.54G (NIH, Bethesda, MD, USA).

### 2.14. Dot Blot Analysis

Secreted proteins in culture supernatant were detected by dot blot [19]. Using a 96-well dot blotter (Carl Roth, Karlsruhe, Germany), 100 µL of supernatant was transferred onto a wet nitrocellulose membrane with a vacuum pump. Ponceau S staining was used for visualization and quantification of total proteins. After being blocked with 5% bovine serum albumin (BSA) in Tris-buffered saline/Tween 20 (TBS-T, 9127.1, Carl Roth, Karlsruhe, Germany) for 1 h, membranes were incubated with primary antibodies at 4 °C overnight. The antibodies used are summarized in Supplementary Table S3. After washing with TBS-T, membranes were exposed to the appropriate secondary antibodies for 2 h. Protein signals were visualized using a chemiluminescence detection system consisting of 100 mM TRIS, 250 mM luminol (Carl Roth, Karlsruhe, Germany), 90 mM p-coumaric acid (Carl Roth, Karlsruhe, Germany), and 30% (*v*/*v*) H_2_O_2_ (Carl Roth, Karlsruhe, Germany). Signal intensities were quantified using ImageJ software.

### 2.15. Western Blot Analysis

The samples were lysed in a freshly prepared RIPA buffer supplemented with protease and phosphatase inhibitors. After quantification with Lowry, proteins (50 µg per sample) were separated by performing sodium dodecyl sulfate–polyacrylamide gel (SDS–PAGE), according to their molecular weight, and transferred to nitrocellulose membranes. Ponceau S staining was used for the visualization of total proteins. After blocking in 5% BSA in TBS-T for 1 h, the membranes were incubated at 4 °C overnight with the primary antibody; the antibodies used are summarized in Table S3. The following day, the membranes were washed by TBS-T, then secondary antibodies were applied for 1 h at room temperature and washed with TBS-T again. For signal development, they were detected by chemiluminescence and quantified by ImageJ [28].

### 2.16. Mineral Content of Bone Scaffold

The mineral content of the bone scaffold was measured using quantitative CT scans on a clinical CT scanner with 128-slice capability. Scanning was conducted with the following settings: 80 kV tube voltage, effective tube current of 500 mA, 16 × 0.3 mm acquisition with a slice thickness of 0.4 mm, and a pitch of 0.4. The images were then reconstructed using a high-resolution V80u kernel and iterative image reconstruction technology at grade 5, and displayed in a bone window. The rectangular axial field-of-view was approximately 10 cm × 10 cm with a matrix resolution of 512 × 512 pixels. The resulting digital imaging and communications in medicine (DICOM) images were imported into ImageJ software via the “DICOM sort” plugin, and the stack was cropped to focus on the region of interest. The mean integrated density for each scaffold was calculated and normalized against the reference block (Phantom EFP-06-96) [38].

### 2.17. Stiffness of Bone Scaffold

Scaffold stiffness was calculated using the Young’s modulus method [19]. The 4 mm scaffolds were compressed four times uniaxially by 10% (speed = 5 mm/min) of the original height by using a ZwickiLine Z 2.5TN material testing machine (ZwickRoell GmbH & Co. KG, Ulm, Germany). An X-force HP 5N sensor was used to measure the applied load in real-time. The load-deformation data were then converted into a stress–strain curve using the height and area of the uncompressed scaffold. Young’s modulus for the region of linear elastic deformation was determined through the following calculation: Young’s modulus [MPa] = (applied force [N] × initial scaffold height [mm])/(area of the scaffold [mm^2^] × change in height [mm]).

### 2.18. Statistical Analysis

The data are presented as means ± the standard error of the mean (SEM). All the experiments were repeated at least three times (*N*) with two or three technical replicates (*n*). Statistical analyses were performed using GraphPad Prism software (GraphPad Software 8.0, La Jolla, CA, USA). The data of the two groups were compared with the Mann–Whitney test. The data of multiple groups were compared with the non-parametric Kruskal–Wallis test, followed by Dunn’s multiple comparison test. A two-way ANOVA test followed by Tukey’s multiple comparisons was used when two independent variables were compared among groups. A *p* < 0.05 was considered statistically significant.

## 3. Results

### 3.1. Long-Term Viability and Functional Maintenance of In Vitro Liver Micro-Organoids for at Least 21 Days

**Figure 2:**
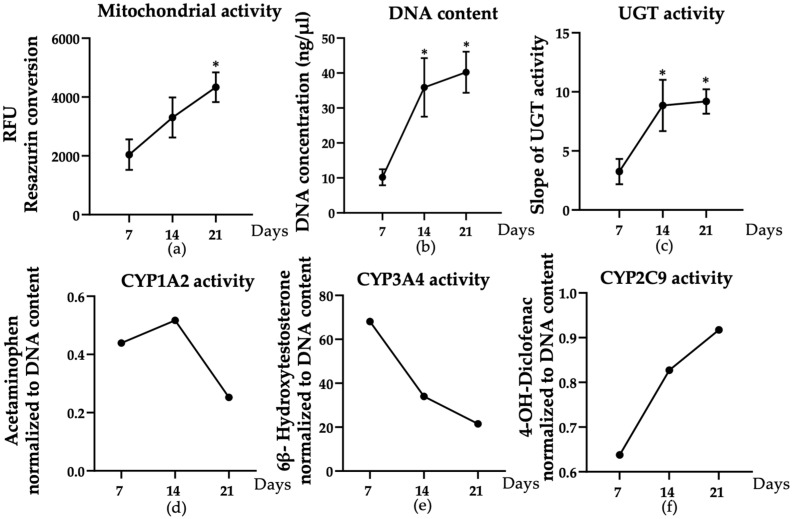
Assessment of long-term stability of the in vitro liver micro-organoids. Differentiated HepaRG cells, LX-2 cells, and HUVECs were used to generate liver micro-organoids at a ratio of 4:2:1 on an agarose plate. Liver micro-organoid viability and function were examined at three different time points (7, 14, and 21 days). Cell viability was detected by (**a**) mitochondrial activity via resazurin conversion (represented as relative fluorescence units (RFU)) and (**b**) DNA quantification. Regarding function, (**c**) UGT activity was normalized to the DNA content as the marker of phase II enzyme activity. The Kruskal–Wallis test followed by Dunn’s multiple comparison test was used to determine statistical differences. Statistical significance is indicated as * *p* < 0.05 vs. the day 7 result. Data are presented as means ± SEM. *N* = 3, *n* = 3. (**d**) CYP1A2 activity, (**e**) CYP3A4 activity, and (**f**) CYP2C9 activity were normalized to DNA content as the markers of phase I enzyme activity from three independent experiments, which pooled the micro-organoids. Data are presented as a line graph. *N* = 3, *n* = 2.

To evaluate the long-term stability of the liver micro-organoids, functional and viability parameters were monitored over a 21-day culture period. Mitochondrial activity, as measured by resazurin reduction, increased continuously throughout the culture (*p* = 0.0454) (Figure 2a), reflecting sustained cellular viability. DNA content also increased significantly by day 14 (*p* = 0.0263) and remained stable thereafter (*p* = 0.0105), indicating cell proliferation and maintenance (Figure 2b). For functional assays, phase II enzyme activity, as assessed by UGT function, increased between days 7 and 14 (*p* = 0.0484) and remained stable until day 21 (*p* = 0.0366) (Figure 2c). Simultaneous measurement of cytochrome P450 basal enzyme activity revealed dynamic changes in enzyme specificity. CYP1A2 activity peaked on day 14 (Figure 2d), while CYP3A4 activity gradually decreased after day 7 but remained at a high level overall compared to levels detected in micro-organoids containing only HepaRG cells [19] (Figure 2e). In contrast, CYP2C9 activity increased steadily throughout the culture period (Figure 2f). Overall, these results indicate that the liver micro-organoids model maintains basic viability and hepatic function over 21 days, supporting its use in long-term in vitro studies.

### 3.2. Induction of Alcohol-Metabolizing Enzyme and Fibrogenic Markers Following Alcohol Exposure in In Vitro Liver Micro-Organoids

**Figure 3:**
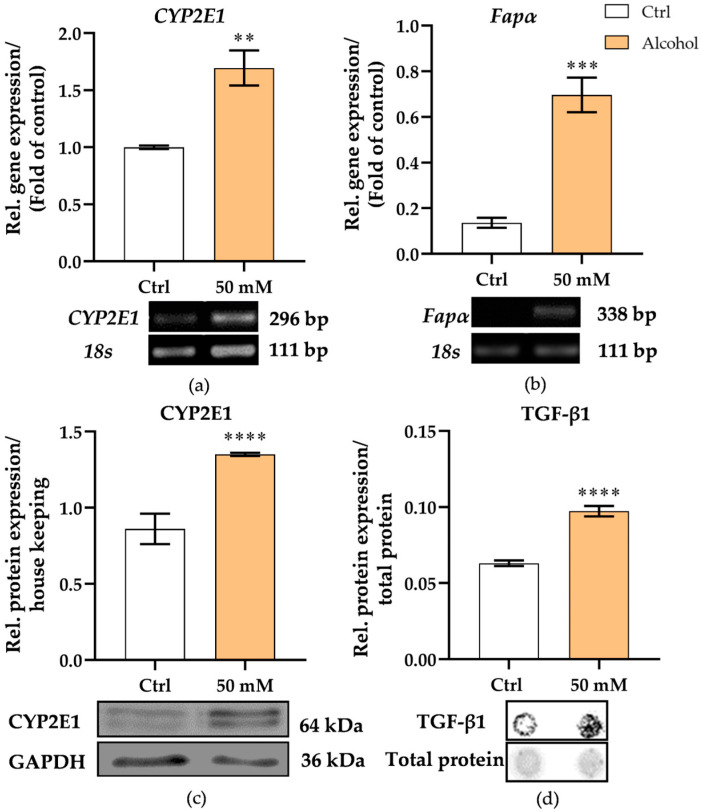
Assessment of alcohol related enzyme expression and stellate cell activation markers. The relative mRNA expression of the key alcohol metabolizing enzyme (**a**) CYP2E1 was detected on day 21. Transcript level of (**b**) *Fapα*, a marker of hepatic stellate cell activation, was measured 48 h after alcohol stimulation. Under the same conditions, the expression of (**c**) CYP2E1 protein in liver micro-organoids and (**d**) the secretion of TGF-β1 protein into the supernatant were also measured on day 21. The Mann–Whitney test was used to determine statistical differences. Statistical significance is indicated as ** *p* < 0.01, *** *p* < 0.001, and **** *p* < 0.0001 vs. the control group. Data are presented as means ± SEM. *N* = 3, *n* ≥ 2.

With the long-term functionality of the liver micro-organoids model established, we then proceeded to assess the alcohol metabolization capacity of liver micro-organoids and the expression of fibrotic markers after daily exposure to alcohol. An alcohol concentration of 50 mM was chosen to stimulate the micro-organoids daily, as this is equivalent to the alcohol intake of four bottles (330 mL) of beer [39], which is considered excessive alcohol consumption. Additionally, daily 50 mM alcohol exposure showed no cytotoxicity on liver micro-organoids (Supplementary Figure S1). After daily 50 mM alcohol treatment, the expression of CYP2E1, which is a key alcohol-metabolizing enzyme in the liver, was significantly increased at both mRNA (*p* = 0.0022) and protein levels (*p* < 0.0001) (Figure 3a,c). Concurrently, the mRNA level of fibroblast activation protein α *(Fapα*), a well-established marker of hepatic stellate cell activation, was significantly upregulated (*p* = 0.0002) (Figure 3b), indicating that LX-2 cells were activated under alcohol stimulation. In addition, alcohol treatment resulted in a significant increase in Transforming Growth Factor Beta 1 (TGF-β1) protein levels (*p* < 0.0001) (Figure 3d), further supporting the initiation of a profibrotic response. These findings suggest that alcohol exposure activates metabolic and profibrotic signaling pathways in the liver micro-organoids.

### 3.3. Alcohol Promotes EMT-Regulating Gene Expression, Matrix-Associated Protein Release, and Liver Damage Markers in Liver Micro-Organoids

**Figure 4:**
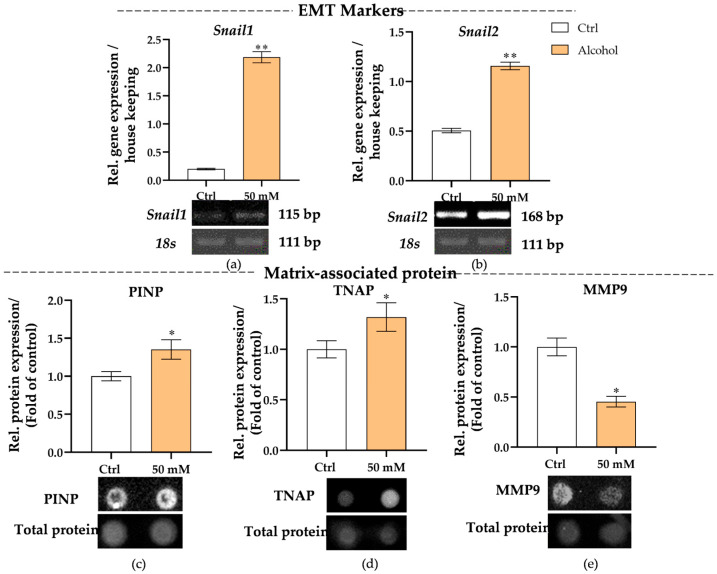
Assessment of early fibrotic-like phenotype in the alcohol-exposed liver micro-organoids. (**a**,**b**) mRNA expression of *Snail* Family Transcriptional Repressor (*Snail1*, *Snail2*), a key transcription factor associated with EMT, was detected at 21 days. (**c**) PINP, a marker of collagen deposition, and (**d**) tissue-nonspecific alkaline phosphatase TNAP was measured on day 21 as an indicator of liver damage or injury. (**e**) Protein levels of MMP9, used as an extracellular matrix degradation, were also measured on day 21. The Mann–Whitney test was used to determine statistical differences. Statistical significance is indicated as * *p* < 0.05, ** *p* < 0.01, vs. the control group. Data are presented as means ± SEM. *N* = 3, *n* = 3.

Previous results have shown that alcohol can be metabolized in the liver micro-organoids and induce the activation of stellate-like cells. To further investigate whether alcohol metabolism in the liver micro-organoids led to pathological remodeling, we examined the expression of epithelial–mesenchymal transition (EMT) markers and extracellular matrix (ECM) related proteins. Gene expression analysis showed that both *Snail1* and *Snail2* were significantly upregulated (*p* = 0.0022) after 21 days of 50 mM daily alcohol stimulation (Figure 4a,b), indicating that EMT-related transcriptional programs were activated. Meanwhile, ECM remodeling was assessed by measuring key matrix-associated proteins in the culture supernatant. The levels of type I procollagen N-terminal propeptide (PINP) in the alcohol-treated group were significantly increased at 21 days (*p* = 0.0379) (Figure 4c). In contrast, the matrix-degrading enzyme matrix metalloproteinase 9 (MMP9) was significantly decreased after alcohol exposure (*p* = 0.0286) (Figure 4e), indicating a decrease in matrix turnover and a shift towards fibrotic matrix accumulation. Additionally, daily alcohol exposure significantly increases the levels of tissue-nonspecific alkaline phosphatase (TNAP) (*p* = 0.0411) (Figure 4d) on liver micro-organoids, indicating injury. These findings, therefore, demonstrate that alcohol exposure in the liver model induces early fibrotic remodeling characterized by EMT activation, altered ECM homeostasis, and liver damage.

### 3.4. Alcohol Does Not Significantly Affect Bone Co-Culture System Function

**Figure 5:**
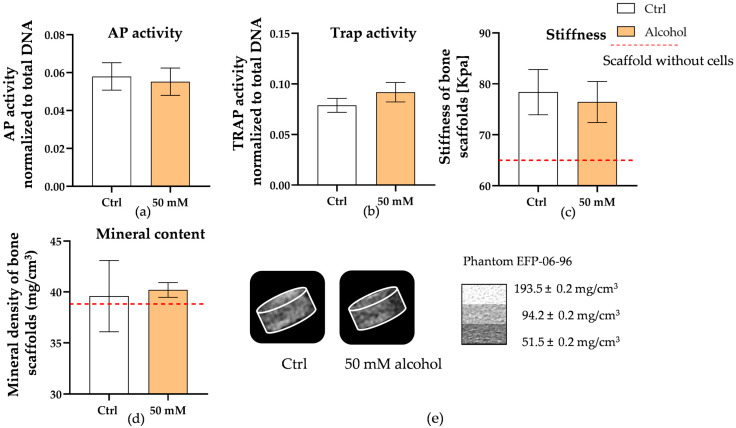
Evaluation of bone co-culture function under 50 mM alcohol exposure. Bone co-culture AP and TRAP activities (**a**,**b**) were measured as functional markers of osteoblasts and osteoclasts, respectively. Bone samples were collected on day 28, and changes in bone homeostasis were evaluated based on stiffness (**c**) and mineral density (**d**). Reconstruction of representative 3D scaffold structures (**e**) was performed following CT scanning.

Before combining the liver and bone system together, we first need to clarify the direct effects of alcohol on the bone co-culture system. We continuously exposed the bone co-culture system to 50 mM alcohol for up to 28 days. Direct exposure to 50 mM alcohol does not affect bone metabolic activity (Supplementary Figure S2). Regarding function, we examined osteoblast activity, which was assessed by AP activity and showed no difference between the alcohol-treated group and the control group on day 21 (Figure 5a). Similarly, osteoclast activity, measured by TRAP assay, did not differ significantly between the control and alcohol-treated groups at the same time point (Figure 5b). This suggests that direct alcohol exposure does not affect the osteogenic and osteoclast functions of the bone co-culture system. To further evaluate the functional outcome of these cellular changes, we measured stiffness and mineral density of the bone scaffolds after 28 days of direct alcohol exposure. Both parameters showed comparable values between the control and alcohol-treated groups (Figure 5c,d). In summary, these findings indicate that direct exposure of the bone co-culture system to 50 mM alcohol did not significantly alter osteoblast and osteoclast activity and function, nor did it significantly affect the overall mechanical properties of the bone scaffold.

### 3.5. Alcohol Exposure Weakens Bone Quality by Inhibiting Mineralization in Liver–Bone Co-Culture System

**Figure 6:**
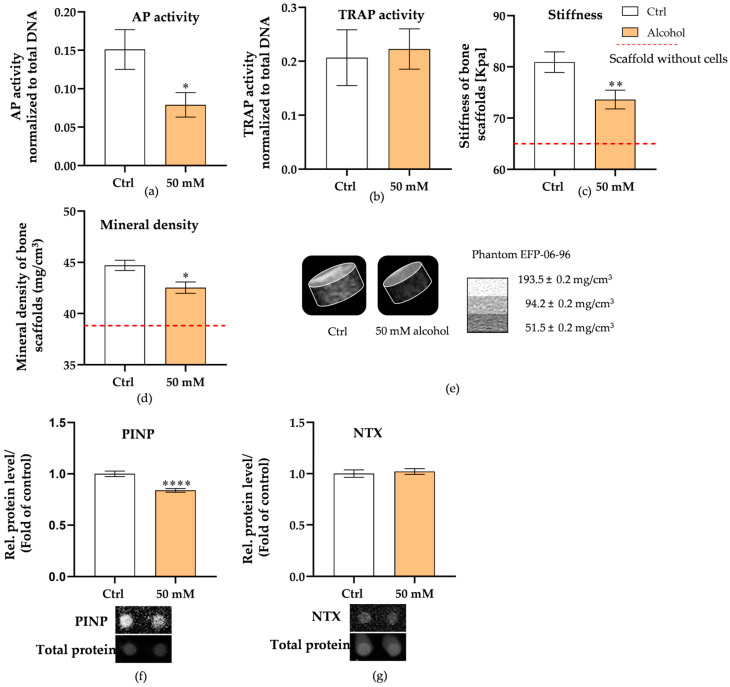
Assessment of bone function under alcohol exposure in the liver–bone co-culture system. (**a**) Osteoblast function was detected using AP activity at 21 days. (**b**) Osteoclast function was detected by TRAP activity at 21 days. (**c**) PINP levels, at 21 days, represent the collagen production. (**d**) NTX levels at 21 days, a marker of collagen degradation. (**e**) Mineral density and (**f**) stiffness tests to measure the changes in bone quality at 28 days. (**g**) Representative 3D reconstructions of the scaffolds obtained by CT scanning are shown. The Mann–Whitney test was used to determine statistical differences. Data are presented as means ± SEM, and the significance is shown as * *p* < 0.05, ** *p* < 0.01, and **** *p* < 0.0001 vs. the control group. *N* = 3, *n* = 3.

Following the confirmation of liver and bone system metabolic activity under alcohol exposure (Supplementary Figure S3), we then assessed the effects of an alcohol-induced pathological liver on bone metabolism. We analyzed key osteoblast and osteoclast markers in the liver–bone co-culture system at day 21. The AP activity was significantly reduced in the alcohol group (*p* = 0.0249) (Figure 6a), indicating impaired bone formation. TRAP activity (Figure 6b) did not differ significantly between the two groups, indicating that osteoclast activity remained stable. Then, we measured scaffold stiffness and bone mineral density. Compared with the control group, 50 mM alcohol treatment significantly reduced the stiffness (*p* = 0.0082) (Figure 6c) and the mineral density (*p* = 0.0117) of the bone scaffold (Figure 6d) after continuous 28-day exposure. To further investigate the effects of alcohol on the bone co-culture system, the PINP protein level in supernatant, a marker of bone collagen synthesis (Figure 6f), was also measured and demonstrated a reduction in the alcohol group (*p* < 0.0001), possibly reflecting the disruption of bone remodeling. The N-terminal telopeptide (NTX) (Figure 6g), a product of collagen degradation that plays a crucial role in bone remodeling and can be used as an indicator of osteoclast activity, showed no significant difference between the two groups. These findings suggest that alcohol-induced liver disease may negatively affect bone metabolism by inhibiting osteoblast function and maintaining osteoclast activity, ultimately leading to impaired bone homeostasis, as demonstrated by a decrease in bone mineral density and bone scaffold stiffness.

### 3.6. Alcohol Exposure Downregulates BMP2 and Upregulates BMP13 in Liver Micro-Organoids

**Figure 7:**
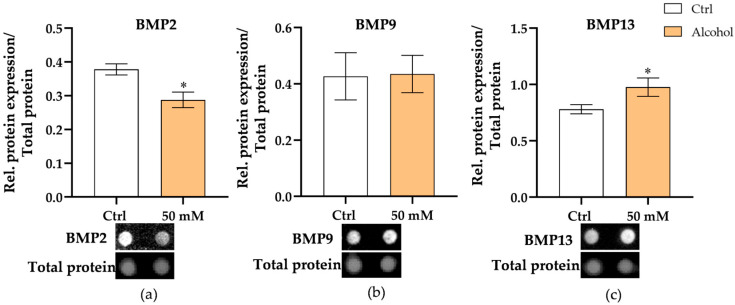
Effect of alcohol on BMPs secretion by liver micro-organoids. Protein levels of BMP2 (**a**), BMP9 (**b**), and BMP13 (**c**) were assessed in control and 50 mM alcohol-treated groups’ cell culture supernatant after 24 h. The protein expression levels were normalized to total protein. The Mann–Whitney test was used to determine statistical differences. Data are presented as means ± SEM, and the significance is shown as * *p* < 0.05 vs. the control group. *N* = 3, *n* = 3.

Based on previous results demonstrating that the deleterious effects of alcohol on bone are predominantly associated with impaired liver function—affecting primarily bone-forming cells—we then investigated how alcohol influences the hepatic secretion profile of BMPs. BMPs were selected for analysis because of the importance of BMP signaling in bone remodeling and osteogenic differentiation, and the existing evidence indicates that their expression patterns are altered under fibrotic conditions [40], suggesting a potential mechanistic link between liver dysfunction and changes in bone homeostasis mediated by these signaling molecules. Since the liver has been reported to secrete BMP2, BMP9, and BMP13 [11,13,41], we measured the BMP protein levels in the culture supernatants. BMP2 levels were significantly decreased in the alcohol group compared to the control (*p* = 0.026) (Figure 7a), while BMP13 levels were significantly increased (*p* = 0.026) (Figure 7c). No significant difference was observed in BMP9 levels between the two groups (Figure 7b). These results suggest that alcohol exposure induces selective alterations in BMP ligand secretion, characterized by reduced BMP2 (promoter of osteogenesis) and increased BMP13 (inhibit osteogenesis) levels, which could shift the balance of progenitor differentiation, impacting bone homeostasis.

### 3.7. Hepatic BMPs Trigger Bone BMP Pathway Activation with Downstream Nuclear Signaling

**Figure 8:**
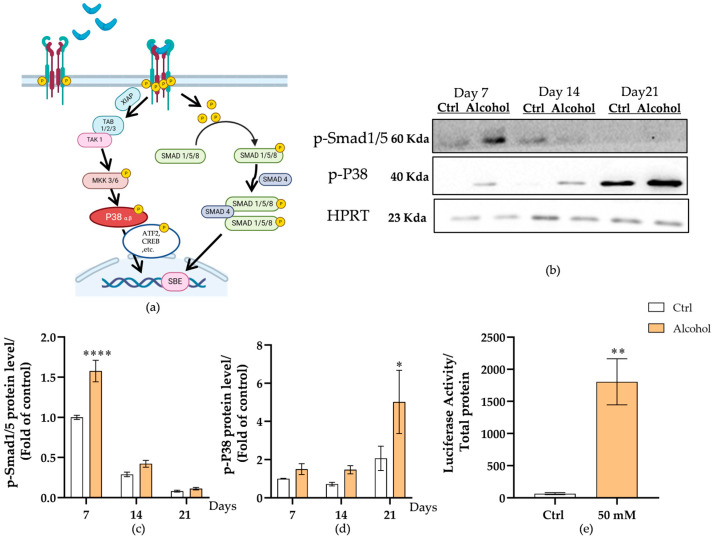
BMP signaling in bone progenitor cells. (**a**) Schematic representation of BMP signaling pathways, illustrating Smad-dependent Smad1/5/8 activation and Smad-independent p38 MAPK signaling. In the Smad-dependent pathway, phosphorylated Smad1, 5, and 8 form heteromeric complexes with Smad4 and translocate into the nucleus. There, they interact with transcriptional cofactors and *RUNX2* to regulate the expression of osteogenic target genes. Alternatively, in the Smad-independent pathway, activated TAK1 recruits TAB1 to initiate downstream signaling cascades, including the MKK-p38 MAPK or MKK-ERK1/2 pathways. These MAPK pathways modulate osteogenic gene expression by phosphorylating and regulating *RUNX2*, thereby influencing osteoblast differentiation. (**b**) Representative immunoblots. HPRT served as the loading control. Quantification of p-Smad1/5 (**c**) and p-P38 (**d**) protein levels at 7, 14, and 21 days post-treatment, expressed as fold of control day 7. (**e**) Luciferase reporter assay demonstrates increased BMP-responsive transcriptional activity in cells treated with alcohol (50 mM) compared to the control. The results were normalized with the total protein amount. The two-way ANOVA followed by Tukey’s multiple comparisons test and Mann–Whitney test were used to determine statistical differences. Data are presented as means ± SEM, and the significance is shown as * *p* < 0.05, ** *p* < 0.01, and **** *p* < 0.0001 vs. the control group. *N* = 3, *n* = 3.

To investigate whether BMP signaling can be properly activated in bone-forming progenitor cells, we examined Smad-dependent and Smad-independent pathways (Figure 8a). Over time, the level of p-Smad1/5 exhibited an overall decreasing trend, while p-P38 showed a progressive increase. When comparing between groups, both p-Smad1/5 and p-P38 levels were consistently higher in the alcohol group than in the control group at all time points (Figure 8b). Quantitative analysis (Figure 8c) further confirmed that p-Smad1/5 levels were elevated in the alcohol group across all time points, especially on day 7 (*p* < 0.0001), whereas p-P38 levels (Figure 8d) demonstrated a time-dependent increase, becoming significantly higher than control at day 21 (*p* = 0.0166). To assess whether alcohol-induced liver-derived factors activated BMP signaling at the nuclear level, we performed a luciferase reporter assay using an adenoviral BMP-responsive element reporter construct (Ad-BRE-Luciferase) in SCP-1 cells. As shown in Figure 8e, luciferase activity was significantly increased in cells treated with the conditioned medium collected from liver micro-organoids exposed to 50 mM alcohol for 24 h compared with cells treated with culture medium from the control group (*p* = 0.0022). This suggests that alcohol-exposed liver micro-organoids secrete BMPs or BMP-inducing factors, which subsequently activate the BMP signaling pathway in SCP-1 cells. The downstream effectors of this pathway translocate to the nucleus of target cells, regulating the differentiation lineage of bone-forming progenitor cells.

### 3.8. Alcohol Impairs Osteogenic Differentiation by Downregulating RUNX2 and Modulating Lineage-Associated Transcription Factors

**Figure 9:**
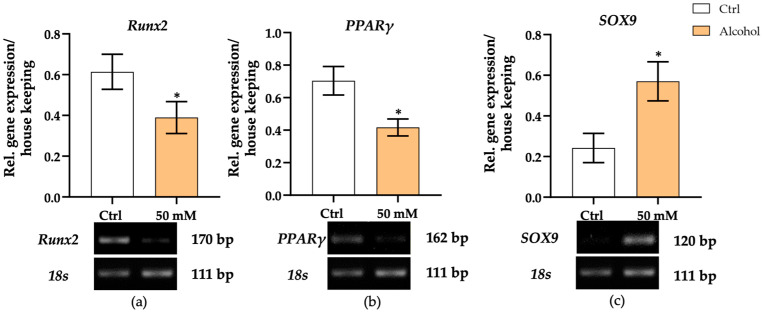
Evaluation of SCP-1 cell lineage-associated transcription factors in the liver–bone system under alcohol stimulation on day 7. To detect changes in the differentiation lineage of SCP-1 cells, the relative expression levels of *RUNX2* (**a**), *PPARγ* (**b**), and *SOX9* (**c**) transcripts were measured after 7 days of the co-culture system with liver micro-organoids under alcohol stimulation. Transcript levels were normalized to the housekeeping gene (18s). The Mann–Whitney test was used to determine statistical differences. Data are presented as means ± SEM, and the significance is shown as * *p* < 0.05 vs. the control group. *N* = 3, *n* = 3.

Since altered secretion levels of BMP2 and BMP13 promoters of osteogenesis and chondrogenesis [16,42], respectively, were detected in the supernatant of liver micro-organoids treated with alcohol, and BMP signaling was shown to occur appropriately in bone-forming progenitor cells, we sought to elucidate the differentiation potential of these progenitors. To achieve this, we analyzed the expression levels of master transcriptional factors involved in osteogenic, adipogenic, and chondrogenic lineages. After 7 days of co-culture under alcohol treatment, RNA was extracted from SCP-1 cells to assess early lineage commitment. Gene expression analysis revealed a significant downregulation of *RUNX2*, a master regulator of osteogenesis, in the alcohol group compared to control (*p* = 0.0315) (Figure 9a). Similarly, *PPARγ*, a transcription factor associated with adipogenic differentiation, was also markedly reduced (*p* = 0.0188) (Figure 9b). In contrast, *SOX9*, a key transcription factor for chondrogenic lineage, showed a significant increase in the alcohol-treated group (*p* = 0.0244) (Figure 9c), suggesting a possible shift in differentiation potential. These results suggest that signals derived from alcohol-exposed liver micro-organoids may disrupt osteogenic and adipogenic programming in SCP-1 cells, while favoring chondrogenic tendencies.

## 4. Discussion

In this study, we successfully developed a long-term in vitro model that mimics alcohol-induced HOD using a 3D co-culture system that integrates liver micro-organoids and bone scaffolds [19]. Now, numerous in vitro studies have investigated the direct effects of alcohol on bone cells, primarily focusing on the inhibitory impact of alcohol on osteoblast proliferation, differentiation, and activity, which ultimately contributes to reduced bone formation and an increased risk of osteoporosis. These studies consistently demonstrate alcohol’s deleterious effects on bone-forming cells, including the induction of oxidative stress, cellular senescence, and impaired mineralization [43,44,45]. However, despite these insights, current in vitro models remain limited as they neglect the potential systemic influence of the liver—a key organ in both alcohol metabolism and the regulation of bone homeostasis. The liver produces and secretes multiple endocrine and paracrine factors, such as BMPs, fibroblast growth factor 21, and TGFβ, all of which critically modulate bone remodeling and cellular function [46,47]. The consideration of liver-derived factors is essential for a comprehensive understanding of alcohol-related skeletal disorders. Integrating hepatic components into bone models enables the investigation of systemic liver–bone interactions and provides mechanistic insight into how hepatic injury contributes to bone deterioration. Our findings highlight the central role of the BMP signaling pathway in mediating alcohol-induced liver–bone crosstalk and suggest that its dysregulation may represent a core mechanism underlying HOD pathogenesis.

In terms of structure, incorporating LX-2 cells and HUVECs into the liver model substantially improves its physiological and pathological relevance compared to HepaRG-only organoids. HepaRG cells supply essential metabolic activity for xenobiotic processing [48] but lack the fibrogenic and vascular components needed to mimic chronic liver disease. Adding LX-2 cells introduces a fibrotic element through extracellular matrix production and pro-fibrogenic signaling [28,49], while the presence of HUVECs enhances vascularization, nutrient exchange, and overall tissue organization [28,50]. Unexpectedly, the liver organoid showed reductions in CYP1A2 and CYP3A4 protein levels, coupled with elevated CYP2C9 activity, which could be explained by the direct protein–protein interactions among these enzymes. Specifically, CYP3A4 engages in heteromeric complex formation with CYP2C9 through hydrophobic interactions at their N-terminal transmembrane domains, potently suppressing CYP2C9 catalytic activity. Dose evidence from CYP3A4 knockdown in human hepatocytes demonstrates that lowering CYP3A4 abundance (~60% reduction) relieves this inhibition, enhancing CYP2C9 activity (~74% increase) independently of changes in CYP2C9 mRNA expression; this effect persists even at saturated cytochrome P450 reductase (CPR) levels, implicating physical docking rather than mere CPR competition. CYP1A2 downregulation parallels CYP3A4 trends and may arise from analogous P450-P450 heteromerization or study-specific factors like oxidative stress, inflammation, or xenobiotic induction patterns, which disproportionately suppress CYP1A2 relative to other isoforms [51,52].

Building upon this framework, selecting an appropriate pathological stimulus is essential for effectively modeling disease-relevant interactions within the liver–bone axis. Alcohol serves as a particularly valuable model for inducing liver fibrosis in the context of HOD research, as it closely replicates the multifactorial, progressive, and systemic nature of human alcoholic liver disease (ALD) [53]. Unlike acute or artificially induced fibrosis models—such as those generated by carbon tetrachloride (CCl_4_) or TGF-β1 administration [28,54]—chronic alcohol exposure-induced hepatic injury not only triggers stellate cell activation and extracellular matrix accumulation but also impairs hepatic endocrine and metabolic function, leading to secondary systemic effects, such as malnutrition, hormonal dysregulation, and low-grade inflammation [55,56]. These mechanisms more accurately reflect the chronic pathological processes observed in patients with ALD and their systemic consequences, including alterations in bone metabolism. By contrast, the CCl_4_ model primarily induces direct hepatocyte toxicity and acute fibrotic scarring, lacking the chronic metabolic and immunological components integral to ALD [54,57]. Similarly, TGF-β-driven fibrosis effectively stimulates fibrogenic signaling but fails to reproduce the nutritional, endocrine, and inflammatory perturbations that profoundly influence bone homeostasis in alcohol-related disease [28]. Moreover, chronic alcohol consumption impairs bone matrix quality through both direct and indirect mechanisms. Directly, ethanol suppresses osteoblast differentiation by inhibiting *Runx2*/Wnt signaling, thereby reducing the synthesis of essential matrix proteins, such as type I collagen, osteocalcin, and osteopontin, while promoting mesenchymal stem cell differentiation into adipocytes, which causes fatty marrow infiltration and defective mineralization. Ethanol also enhances osteoclastogenesis via RANKL upregulation, increasing bone resorption. Indirectly, alcohol-induced liver dysfunction disrupts vitamin D activation and IGF-1 production—critical regulators of osteoblast function and matrix deposition—leading to reduced growth factor availability in bone and greater matrix fragility. Furthermore, alcohol promotes hypocalcemia through intestinal malabsorption and vitamin D deficiency, compounded by hypercortisolemia and hypogonadism, which lower estrogen/testosterone levels necessary for bone maintenance, ultimately shifting remodeling toward net resorption and accelerating osteopenia.

Accordingly, we combined the liver and bone components to generate a liver–bone co-culture system, examining how alcohol-induced liver fibrosis perturbs bone homeostasis. Previous studies have reported divergent mechanisms underlying alcohol-induced bone loss. Some suggest that alcohol promotes osteoclastogenesis via RANKL-mediated pathways, often as a result of elevated inflammatory signaling [58,59]. By contrast, other studies emphasize that the dominant effect of alcohol is the suppression of osteoblast differentiation and function, rather than a direct enhancement of osteoclastic activity [60,61]. Our findings are consistent with the latter mechanism: despite prolonged alcohol exposure, osteoclast function remained unchanged, while osteoblast activity, mineral deposition, and *RUNX2* expression were significantly reduced. These results underscore the importance of targeting osteoblast dysfunction in future therapeutic strategies for alcohol-related bone disease. However, several factors may contribute to the lack of observable osteoclast activation in our system. As shown in previous studies, inflammatory factors are the main cause of osteoclast functional alterations. However, our liver micro-organoids lack Kupffer cells and therefore cannot produce large amounts of inflammatory cytokines in response to external stimuli as a normal liver does. The absence of Kupffer cells underestimates inflammatory contributions to the liver–bone axis; their omission limits recapitulation of macrophage-driven fibrogenesis observed clinically. Future studies need to incorporate Kupffer cells or iPSC-derived macrophage co-cultures to comprehensively model innate immunity’s role in HOD. Furthermore, alcohol-induced changes in osteoclast function may be a secondary consequence of impaired osteoblast activity rather than a direct effect, and such changes might require prolonged exposure, higher alcohol concentrations, or the presence of inflammatory mediators to manifest [62].

This selective suppression of osteogenesis, in the absence of overt osteoclast activation, prompted us to prioritize pathways governing mesenchymal lineage allocation—most notably the BMP axis [63]. BMPs exert multiple effects in promoting the differentiation of bone mesenchymal stem cells (BMCs) toward osteogenic, adipogenic, or chondrogenic lineages [64,65]. The cellular and therapeutic actions of BMPs are mediated through downstream signaling cascades that are initiated when BMPs bind to transmembrane serine/threonine kinase receptors. This interaction activates specific intracellular signaling pathways—primarily the SMAD-dependent and SMAD-independent routes—that regulate transcription programs involved in cell differentiation and tissue development [66]. Previous studies have demonstrated that during pathological liver remodeling, the expression and secretion of several BMP family members, including BMP2, BMP9, and BMP13, are markedly altered [13,41,67]. Notably, BMP2 expression tends to decrease following hepatic fibrosis. This reduction may be attributed to the loss of hepatocyte function and the replacement of parenchymal tissue with extracellular matrix components, which limits the capacity of the liver to produce osteogenic cytokines [67]. In contrast, BMP9, which is mainly produced by hepatic sinusoidal endothelial cells, often exhibits a more complex regulatory pattern—its expression may transiently increase during early injury to support vascular remodeling but subsequently decline as fibrosis progresses [12]. BMP13, another member associated with fibrogenic processes, has been reported to be upregulated in activated hepatic stellate cells, suggesting its potential involvement in extracellular matrix deposition and tissue stiffness regulation [13]. These differential changes in BMP expression imply that liver pathology may not only alter local hepatic signaling but also influence systemic BMP availability. Since BMP2, BMP9, and BMP13 are key regulators of BMCs differentiation, such hepatic alterations could indirectly affect bone remodeling and regeneration. Our findings, therefore, highlight a possible link between liver injury-induced BMP dysregulation and bone homeostasis under pathological conditions. Our results suggest that alcohol disrupts bone homeostasis by differentially regulating BMP secretion (↓BMP2 and↑BMP13). Although BMP2 and BMP13 (GDF6) both activate the Smad1/5/8 pathway via type I BMP receptors, they differ in receptor affinity and functional outcomes. BMP2 binds both ALK3 and ALK6 with similar affinity, promoting robust osteogenic differentiation across cell types [68,69]. In contrast, BMP13 preferentially binds ALK6 [69,70], limiting its signaling to specific cells and favoring tenogenic or chondrogenic programs. By competing for type II receptors without efficiently activating ALK3, BMP13 may also antagonize BMP2 activity. These distinctions in receptor selectivity, target activation, and tissue distribution provide a molecular basis for cell fate divergence.

Compared with existing studies, several in vivo studies have reported that alcohol exposure can impair BMP2 function by inhibiting BMP receptor activity [67]. Furthermore, some studies have demonstrated that the overall expression level of BMP2 is significantly decreased during the development of fibrosis [11,71]. Alcohol-induced inhibition of BMP2 may not only hinder liver tissue repair but also disrupt bone remodeling, contributing to decreased bone density through the liver–bone axis. In contrast, BMP13 appears to be upregulated during liver fibrosis, although relevant studies remain limited [41,72]. Consistent with these reports, our model also showed similar BMP secretion patterns, supporting its reliability. Further analysis of protein-level signaling revealed that phosphorylated Smad1/5 (p-Smad1/5) levels increased significantly on day 7, coinciding with the upregulation of *SOX9*. These results suggest that alcohol may initiate chondrogenic gene programs via the BMP13–Smad1/5–*SOX9* signaling axis, rather than promoting osteogenic differentiation through the SMAD-dependent BMP2–*RUNX2* pathway. In contrast, phosphorylated p38 (p-p38) expression did not show a significant increase on day 7 but gradually rose by day 21, suggesting it may function as a regulatory factor in maintaining *SOX9* expression or promoting chondrocyte maturation at later stages. Although we did not observe a change in total BMP9, prior studies indicate that biological activity depends on the proportion of dimeric, disulfide-stabilized forms [73]. Chronic alcohol exposure can perturb protein folding pathways in hepatocytes and might thereby influence BMP9 bioactivity despite stable abundance.

The temporal dynamics of the BMP-signaling-pathway components revealed distinct regulatory patterns in our system. The SMAD-dependent component (p-Smad1/5) declined over time in both groups, consistent with its early role in lineage commitment and subsequent downregulation during cell maturation [74]. However, its levels are downregulated as cells enter maturation and matrix production phases. In contrast, p-p38 levels increased over time and was further enhanced by alcohol, likely reflecting stress and inflammatory responses in the liver–bone co-culture, since this SMAD-independent BMP branch is known to respond to oxidative stress and inflammatory cytokines [75]. Since p-p38 can promote *SOX9* expression, this shift may redirect differentiation toward the chondrogenic lineage [76]. Overall, alcohol appears to suppress osteogenic signaling while activating stress-related BMP pathways, contributing to impaired MSC differentiation and bone loss.

Both osteogenesis and adipogenesis are metabolically demanding processes that rely heavily on mitochondrial integrity and oxidative phosphorylation [77]. Alcohol metabolism, primarily via hepatic CYP2E1 activity, leads to the generation of reactive oxygen species, which impairs mitochondrial membrane potential, ATP production, and redox balance [78,79]. These metabolic disruptions can inhibit master transcription factors such as *RUNX2* and *PPARγ*, thereby suppressing osteoblast and adipocyte lineage commitment. In contrast, chondrogenesis, regulated by *SOX9*, is comparatively less dependent on mitochondrial function and is often upregulated under hypoxic, low-energy, or inflammatory conditions. Moreover, systemic inflammatory signals from the liver, including elevated IL-6 and TNF-α, may selectively inhibit osteogenic and adipogenic programs while permissively supporting fibrocartilage differentiation [80,81,82]. Additionally, the antagonistic interaction between *SOX9* and *RUNX2* may further amplify this fate divergence, as the inhibition of osteogenesis can indirectly favor chondrogenic dominance [83]. The enhanced chondrogenesis may reflect a stress-adaptive response, as mesenchymal stem cells under oxidative or inflammatory stress tend to favor the chondrogenic lineage for its lower energy demand and protective matrix production [78].

These mechanistic insights have important implications for the treatment of alcohol-induced bone disorders, particularly HOD, where conventional bone-targeted therapies may not sufficiently address the underlying systemic drivers. While standard approaches to treating bone loss typically involve the use of osteoanabolic agents or antiresorptive drugs [84]—such as parathyroid hormone analogs, vitamin D supplementation, or bisphosphonates—our findings suggest that such interventions may be insufficient in the context of HOD. In this condition, bone dysfunction is primarily driven by chronic liver injury and its systemic consequences, including altered cytokine secretion profile, oxidative stress, and metabolic imbalances. Therefore, achieving long-term improvement in bone health requires addressing the underlying hepatic pathology. Therapeutic strategies aimed at reducing liver inflammation and oxidative stress—such as antioxidants and anti-inflammatory agents—may help limit the production of bone-damaging mediators [85]. Additionally, restoring hepatocyte function and promoting hepatic repair could indirectly support bone remodeling by rebalancing liver–bone signaling axes, including IGF-1, HGF, and FGF pathways.

A key strength of this study is the novel 3D liver–bone co-culture system, which captures inter-organ BMP signaling and pathological crosstalk unattainable in monoculture or single-organoid models. However, the 28-day alcohol exposure, while physiologically relevant (50 mM ≈ 4 beers/day), cannot fully replicate decades-long human consumption patterns. Additionally, the static in vitro setup lacks mechanical loading, vascular perfusion, and systemic endocrine/hormonal influences present in vivo, which should be considered when extrapolating these findings to clinical hepatic osteodystrophy. Future studies incorporating dynamic bioreactors and multi-organ chips will be valuable to address these gaps.

## 5. Conclusions

In summary, we successfully established a long-term human in vitro liver–bone co-culture model that mimics key features of alcohol-induced HOD. We established a physiologically relevant, long-term human 3D in vitro liver–bone co-culture system that integrates key hepatic cell types (hepatocytes, hepatic stellate cells, and endothelial cells) with bone cell types (osteoblasts and osteoclasts). This model mimics the dynamic and reciprocal interactions between the liver and bone under chronic alcohol exposure. Compared to previous studies, it offers enhanced control over cellular composition, microenvironment, and exposure duration, making it well-suited for investigating the mechanistic basis of HOD. Furthermore, this platform offers a unique opportunity to investigate inter-organ signaling mechanisms, with a particular focus on BMP-mediated communication, which plays a central role in osteoblast differentiation. Our findings highlight the central role of BMP imbalance—specifically, the downregulation of BMP2 and upregulation of BMP13—in mediating the shift from osteogenic to chondrogenic lineage commitment. This mechanistic insight, coupled with the controllability and translational potential of our in vitro platform, provides a valuable tool for studying inter-organ metabolic crosstalk in CLD. In addition, this model aligns with the 3R principles by reducing dependence on animal experiments and offering an ethical, scalable platform for therapeutic screening.

## Data Availability

The original contributions presented in the study are included in the article. Further inquiries can be directed to the corresponding author.

## Supplementary Materials

The following supporting information can be downloaded at: https://www.mdpi.com/article/10.3390/cells15030274/s1, Table S1: List of different medium, Table S2: Human primer sequences used in PCR, Table S3: List of antibodies used for Western blot, Figure S1: Alcohol toxicity test in 3D liver micro-organoids on day 7, Figure S2: Alcohol toxicity test in the 3D bone co-culture system, Figure S3: Viability of bone and liver micro-organoids in the liver–bone co-culture system.

## Abbreviations

The following abbreviations are used in this manuscript:

AP	Alkaline phosphatase
ALD	Alcoholic liver disease
BMPs	Bone morphogenetic proteins
BMCs	Bone mesenchymal stem cells
CLD	Chronic liver disease
CYPs	Cytochrome p450
DMSO	Dimethyl sulfoxide
FCS	Fetal calf serum
HSCs	Hepatic stellate cells
HOD	Hepatic osteodystrophy
hPRP	Human platelet-rich plasma
GLU	Glutamine
LDH	Lactate dehydrogenase
MMP9	Matrix metalloproteinase-9
MSCs	Mesenchymal stem cells
NTX	N-terminal telopeptide
P/S	Penicillin/streptomycin
PRP	Platelet-rich plasma
TRAP	Tartrate-resistant acid phosphatase
TNAP	Non-specific alkaline phosphatase
UGT	UDP-glucuronosyltransferase

## References

[1] Ehnert S., Aspera-Werz R.H., Ruoß M., Dooley S., Hengstler J.G., Nadalin S., Relja B., Badke A., Nussler A.K. (2019). Hepatic Osteodystrophy-Molecular Mechanisms Proposed to Favor Its Development. Int. J. Mol. Sci..

[2] Collier J. (2007). Bone disorders in chronic liver disease. Hepatology.

[3] Lu K., Shi T.S., Shen S.Y., Shi Y., Gao H.L., Wu J., Lu X., Gao X., Ju H.X., Wang W. (2022). Defects in a liver-bone axis contribute to hepatic osteodystrophy disease progression. Cell Metab..

[4] Gao H., Peng X., Li N., Gou L., Xu T., Wang Y., Qin J., Liang H., Ma P., Li S. (2024). Emerging role of liver-bone axis in osteoporosis. J. Orthop. Transl..

[5] Osna N.A., Donohue T.M., Kharbanda K.K. (2017). Alcoholic Liver Disease: Pathogenesis and Current Management. Alcohol Res..

[6] Nagarjuna D., Karthikeyan E. (2025). Alcohol-associated liver disease: A review. Gastroenterol. Endosc..

[7] World Health Organization. Regional Office for Europe (2024). Alcohol Taxation and Pricing Policies and Their Impact on Alcohol Consumption in Ukraine from 2011–2021: Country Report.

[8] World Health Organization Global Information System on Alcohol and Health. Levels of Consumption. https://www.who.int/data/gho/data/themes/topics/topic-details/GHO/levels-of-consumption.

[9] Page A., Paoli P.P., Hill S.J., Howarth R., Wu R., Kweon S.M., French J., White S., Tsukamoto H., Mann D.A. (2015). Alcohol directly stimulates epigenetic modifications in hepatic stellate cells. J. Hepatol..

[10] Patidar P., Hirani N., Bharti S., Baig M.S. (2024). Key regulators of hepatic stellate cell activation in alcohol liver Disease: A comprehensive review. Int. Immunopharmacol..

[11] Chung Y.H., Huang Y.H., Chu T.H., Chen C.L., Lin P.R., Huang S.C., Wu D.C., Huang C.C., Hu T.H., Kao Y.H. (2018). BMP-2 restoration aids in recovery from liver fibrosis by attenuating TGF-β1 signaling. Lab. Investig..

[12] Desroches-Castan A., Tillet E., Ricard N., Ouarné M., Mallet C., Belmudes L., Couté Y., Boillot O., Scoazec J.Y., Bailly S. (2019). Bone Morphogenetic Protein 9 Is a Paracrine Factor Controlling Liver Sinusoidal Endothelial Cell Fenestration and Protecting Against Hepatic Fibrosis. Hepatology.

[13] Peschl V., Seitz T., Sommer J., Thasler W., Bosserhoff A., Hellerbrand C. (2022). Bone morphogenetic protein 13 in hepatic stellate cells and hepatic fibrosis. J. Cell. Biochem..

[14] Bordukalo-Nikšić T., Kufner V., Vukičević S. (2022). The Role Of BMPs in the Regulation of Osteoclasts Resorption and Bone Remodeling: From Experimental Models to Clinical Applications. Front. Immunol..

[15] Zhu D., Mackenzie N.C., Shanahan C.M., Shroff R.C., Farquharson C., MacRae V.E. (2015). BMP-9 regulates the osteoblastic differentiation and calcification of vascular smooth muscle cells through an ALK1 mediated pathway. J. Cell. Mol. Med..

[16] Shen B., Bhargav D., Wei A., Williams L.A., Tao H., Ma D.D., Diwan A.D. (2009). BMP-13 emerges as a potential inhibitor of bone formation. Int. J. Biol. Sci..

[17] Ponnappa B.C., Rubin E. (2000). Modeling alcohol’s effects on organs in animal models. Alcohol Res. Health.

[18] Osyczka A.M., Diefenderfer D.L., Bhargave G., Leboy P.S. (2004). Different effects of BMP-2 on marrow stromal cells from human and rat bone. Cells Tissues Organs.

[19] Chen G., Xin Y., Hammour M.M., Braun B., Ehnert S., Springer F., Vosough M., Menger M.M., Kumar A., Nüssler A.K. (2025). Establishment of a human 3D in vitro liver-bone model as a potential system for drug toxicity screening. Arch. Toxicol..

[20] Moon A.M., Singal A.G., Tapper E.B. (2020). Contemporary Epidemiology of Chronic Liver Disease and Cirrhosis. Clin. Gastroenterol. Hepatol..

[21] Karlsen T.H., Sheron N., Zelber-Sagi S., Carrieri P., Dusheiko G., Bugianesi E., Pryke R., Hutchinson S.J., Sangro B., Martin N.K. (2022). The EASL–*Lancet* Liver Commission: Protecting the next generation of Europeans against liver disease complications and premature mortality. Lancet.

[22] Prince D.S., Nash E., Liu K. (2023). Alcohol-Associated Liver Disease: Evolving Concepts and Treatments. Drugs.

[23] Jang M., Kleber A., Ruckelshausen T., Betzholz R., Manz A. (2019). Differentiation of the human liver progenitor cell line (HepaRG) on a microfluidic-based biochip. J. Tissue Eng. Regen. Med..

[24] Hong Y., Li S., Wang J., Li Y. (2018). In vitro inhibition of hepatic stellate cell activation by the autophagy-related lipid droplet protein ATG2A. Sci. Rep..

[25] Song Y., Kim S., Heo J., Shum D., Lee S.-Y., Lee M., Kim A.R., Seo H.R. (2021). Identification of hepatic fibrosis inhibitors through morphometry analysis of a hepatic multicellular spheroids model. Sci. Rep..

[26] Böcker W., Yin Z., Drosse I., Haasters F., Rossmann O., Wierer M., Popov C., Locher M., Mutschler W., Docheva D. (2008). Introducing a single-cell-derived human mesenchymal stem cell line expressing hTERT after lentiviral gene transfer. J. Cell. Mol. Med..

[27] Ghezelayagh Z., Zabihi M., Zarkesh I., Gonçalves C.A.C., Larsen M., Hagh-parast N., Pakzad M., Vosough M., Arjmand B., Baharvand H. (2022). Improved Differentiation of hESC-Derived Pancreatic Progenitors by Using Human Fetal Pancreatic Mesenchymal Cells in a Micro-scalable Three-Dimensional Co-culture System. Stem Cell Rev. Rep..

[28] Zahmatkesh E., Othman A., Braun B., Aspera R., Ruoß M., Piryaei A., Vosough M., Nüssler A. (2022). In vitro modeling of liver fibrosis in 3D microtissues using scalable micropatterning system. Arch. Toxicol..

[29] Häussling V., Deninger S., Vidoni L., Rinderknecht H., Ruoß M., Arnscheidt C., Athanasopulu K., Kemkemer R., Nussler A.K., Ehnert S. (2019). Impact of Four Protein Additives in Cryogels on Osteogenic Differentiation of Adipose-Derived Mesenchymal Stem Cells. Bioengineering.

[30] Weng W., Häussling V., Aspera-Werz R.H., Springer F., Rinderknecht H., Braun B., Küper M.A., Nussler A.K., Ehnert S. (2020). Material-Dependent Formation and Degradation of Bone Matrix-Comparison of Two Cryogels. Bioengineering.

[31] Häussling V., Aspera-Werz R.H., Rinderknecht H., Springer F., Arnscheidt C., Menger M.M., Histing T., Nussler A.K., Ehnert S. (2021). 3D Environment Is Required In Vitro to Demonstrate Altered Bone Metabolism Characteristic for Type 2 Diabetics. Int. J. Mol. Sci..

[32] Hammour M.M., Othman A., Aspera-Werz R., Braun B., Weis-Klemm M., Wagner S., Nadalin S., Histing T., Ruoß M., Nüssler A.K. (2022). Optimisation of the HepaRG cell line model for drug toxicity studies using two different cultivation conditions: Advantages and limitations. Arch. Toxicol..

[33] Aspera-Werz R.H., Ehnert S., Heid D., Zhu S., Chen T., Braun B., Sreekumar V., Arnscheidt C., Nussler A.K. (2018). Nicotine and Cotinine Inhibit Catalase and Glutathione Reductase Activity Contributing to the Impaired Osteogenesis of SCP-1 Cells Exposed to Cigarette Smoke. Oxidative Med. Cell. Longev..

[34] Guo H., Weng W., Zhang S., Rinderknecht H., Braun B., Breinbauer R., Gupta P., Kumar A., Ehnert S., Histing T. (2022). Maqui Berry and Ginseng Extracts Reduce Cigarette Smoke-Induced Cell Injury in a 3D Bone Co-Culture Model. Antioxidants.

[35] Zhu S., Häussling V., Aspera-Werz R.H., Chen T., Braun B., Weng W., Histing T., Nussler A.K. (2021). Bisphosphonates Reduce Smoking-Induced Osteoporotic-Like Alterations by Regulating RANKL/OPG in an Osteoblast and Osteoclast Co-Culture Model. Int. J. Mol. Sci..

[36] Godel M., Morena D., Ananthanarayanan P., Buondonno I., Ferrero G., Hattinger C.M., Di Nicolantonio F., Serra M., Taulli R., Cordero F. (2020). Small Nucleolar RNAs Determine Resistance to Doxorubicin in Human Osteosarcoma. Int. J. Mol. Sci..

[37] Aspera-Werz R.H., Chen T., Ehnert S., Zhu S., Fröhlich T., Nussler A.K. (2019). Cigarette Smoke Induces the Risk of Metabolic Bone Diseases: Transforming Growth Factor Beta Signaling Impairment via Dysfunctional Primary Cilia Affects Migration, Proliferation, and Differentiation of Human Mesenchymal Stem Cells. Int. J. Mol. Sci..

[38] Fu W., Huo R., Yan Z., Xu H., Li H., Jiao Y., Wang L., Weng J., Wang J., Wang S. (2020). Mesenchymal Behavior of the Endothelium Promoted by SMAD6 Downregulation Is Associated with Brain Arteriovenous Malformation Microhemorrhage. Stroke.

[39] Jones A.W., Holmgren P. (2003). Comparison of blood-ethanol concentration in deaths attributed to acute alcohol poisoning and chronic alcoholism. J. Forensic Sci..

[40] Herrera B., Addante A., Sánchez A. (2018). BMP Signalling at the Crossroad of Liver Fibrosis and Regeneration. Int. J. Mol. Sci..

[41] Chen H., Li Y.Y., Nio K., Tang H. (2024). Unveiling the Impact of BMP9 in Liver Diseases: Insights into Pathogenesis and Therapeutic Potential. Biomolecules.

[42] Cai H., Zou J., Wang W., Yang A. (2021). BMP2 induces hMSC osteogenesis and matrix remodeling. Mol. Med. Rep..

[43] Wang Y., Li Y., Mao K., Li J., Cui Q., Wang G.-J. (2003). Alcohol-Induced Adipogenesis in Bone and Marrow: A Possible Mechanism for Osteonecrosis. Clin. Orthop. Relat. Res.^®^.

[44] Lewis S.A., Doratt B.M., Qiao Q., Blanton M., Grant K.A., Messaoudi I. (2023). Integrated single cell analysis shows chronic alcohol drinking disrupts monocyte differentiation in the bone marrow. Stem Cell Rep..

[45] Cui Q., Wang Y., Saleh K.J., Wang G.J., Balian G. (2006). Alcohol-induced adipogenesis in a cloned bone-marrow stem cell. J. Bone Jt. Surg. Am..

[46] Wang X., Wei W., Krzeszinski J.Y., Wang Y., Wan Y. (2015). A Liver-Bone Endocrine Relay by IGFBP1 Promotes Osteoclastogenesis and Mediates FGF21-Induced Bone Resorption. Cell Metab..

[47] He T., Qin L., Chen S., Huo S., Li J., Zhang F., Yi W., Mei Y., Xiao G. (2025). Bone-derived factors mediate crosstalk between skeletal and extra-skeletal organs. Bone Res..

[48] DiProspero T.J., Brown L.G., Fachko T.D., Lockett M.R. (2022). HepaRG cells adopt zonal-like drug-metabolizing phenotypes under physiologically relevant oxygen tensions and Wnt/β-catenin signaling. Drug Metab. Dispos..

[49] Zhang C.Y., Yuan W.G., He P., Lei J.H., Wang C.X. (2016). Liver fibrosis and hepatic stellate cells: Etiology, pathological hallmarks and therapeutic targets. World J. Gastroenterol..

[50] Kocherova I., Bryja A., Mozdziak P., Angelova Volponi A., Dyszkiewicz-Konwińska M., Piotrowska-Kempisty H., Antosik P., Bukowska D., Bruska M., Iżycki D. (2019). Human Umbilical Vein Endothelial Cells (HUVECs) Co-Culture with Osteogenic Cells: From Molecular Communication to Engineering Prevascularised Bone Grafts. J. Clin. Med..

[51] Subramanian M., Tam H., Zheng H., Tracy T.S. (2010). CYP2C9-CYP3A4 protein-protein interactions: Role of the hydrophobic N terminus. Drug Metab. Dispos..

[52] Ramsden D., Tweedie D.J., Chan T.S., Tracy T.S. (2014). Altered CYP2C9 activity following modulation of CYP3A4 levels in human hepatocytes: An example of protein-protein interactions. Drug Metab. Dispos..

[53] Hong X., Huang S., Jiang H., Ma Q., Qiu J., Luo Q., Cao C., Xu Y., Chen F., Chen Y. (2024). Alcohol-related liver disease (ALD): Current perspectives on pathogenesis, therapeutic strategies, and animal models. Front. Pharmacol..

[54] Nussler A.K., Wildemann B., Freude T., Litzka C., Soldo P., Friess H., Hammad S., Hengstler J.G., Braun K.F., Trak-Smayra V. (2014). Chronic CCl4 intoxication causes liver and bone damage similar to the human pathology of hepatic osteodystrophy: A mouse model to analyse the liver-bone axis. Arch. Toxicol..

[55] Yang L., Latchoumycandane C., McMullen M.R., Pratt B.T., Zhang R., Papouchado B.G., Nagy L.E., Feldstein A.E., McIntyre T.M. (2010). Chronic alcohol exposure increases circulating bioactive oxidized phospholipids. J. Biol. Chem..

[56] Åberg F., Byrne C.D., Pirola C.J., Männistö V., Sookoian S. (2023). Alcohol consumption and metabolic syndrome: Clinical and epidemiological impact on liver disease. J. Hepatol..

[57] Fujii T., Fuchs B.C., Yamada S., Lauwers G.Y., Kulu Y., Goodwin J.M., Lanuti M., Tanabe K.K. (2010). Mouse model of carbon tetrachloride induced liver fibrosis: Histopathological changes and expression of CD133 and epidermal growth factor. BMC Gastroenterol..

[58] Dai J., Lin D., Zhang J., Habib P., Smith P., Murtha J., Fu Z., Yao Z., Qi Y., Keller E.T. (2000). Chronic alcohol ingestion induces osteoclastogenesis and bone loss through IL-6 in mice. J. Clin. Investig..

[59] de Almeida J.M., Pazmino V.F.C., Novaes V.C.N., Bomfim S.R.M., Nagata M.J.H., Oliveira F.L.P., Matheus H.R., Ervolino E. (2020). Chronic consumption of alcohol increases alveolar bone loss. PLoS ONE.

[60] Dyer S.A., Buckendahl P., Sampson H.W. (1998). Alcohol consumption inhibits osteoblastic cell proliferation and activity in vivo. Alcohol.

[61] Guo M., Huang Y.L., Wu Q., Chai L., Jiang Z.Z., Zeng Y., Wan S.R., Tan X.Z., Long Y., Gu J.L. (2021). Chronic Ethanol Consumption Induces Osteopenia via Activation of Osteoblast Necroptosis. Oxidative Med. Cell. Longev..

[62] Kamiya N., Kobayashi T., Mochida Y., Yu P.B., Yamauchi M., Kronenberg H.M., Mishina Y. (2010). Wnt inhibitors Dkk1 and Sost are downstream targets of BMP signaling through the type IA receptor (BMPRIA) in osteoblasts. J. Bone Miner. Res..

[63] Wu Z., Li W., Jiang K., Lin Z., Qian C., Wu M., Xia Y., Li N., Zhang H., Xiao H. (2024). Regulation of bone homeostasis: Signaling pathways and therapeutic targets. MedComm.

[64] Beederman M., Lamplot J.D., Nan G., Wang J., Liu X., Yin L., Li R., Shui W., Zhang H., Kim S.H. (2013). BMP signaling in mesenchymal stem cell differentiation and bone formation. J. Biomed. Sci. Eng..

[65] Kang Q., Song W.X., Luo Q., Tang N., Luo J., Luo X., Chen J., Bi Y., He B.C., Park J.K. (2009). A comprehensive analysis of the dual roles of BMPs in regulating adipogenic and osteogenic differentiation of mesenchymal progenitor cells. Stem Cells Dev..

[66] Zhang J., Li L. (2005). BMP signaling and stem cell regulation. Dev. Biol..

[67] Gerjevic L.N., Liu N., Lu S., Harrison-Findik D.D. (2012). Alcohol Activates TGF-Beta but Inhibits BMP Receptor-Mediated Smad Signaling and Smad4 Binding to Hepcidin Promoter in the Liver. Int. J. Hepatol..

[68] Jang W.G., Kim E.J., Kim D.K., Ryoo H.M., Lee K.B., Kim S.H., Choi H.S., Koh J.T. (2012). BMP2 protein regulates osteocalcin expression via *Runx2*-mediated Atf6 gene transcription. J. Biol. Chem..

[69] Berasi S.P., Varadarajan U., Archambault J., Cain M., Souza T.A., Abouzeid A., Li J., Brown C.T., Dorner A.J., Seeherman H.J. (2011). Divergent activities of osteogenic BMP2, and tenogenic BMP12 and BMP13 independent of receptor binding affinities. Growth Factors.

[70] Lin S., Svoboda K.K.H., Feng J.Q., Jiang X. (2016). The biological function of type I receptors of bone morphogenetic protein in bone. Bone Res..

[71] Yang M., Zhang C. (2021). The role of bone morphogenetic proteins in liver fibrosis. Gastroenterol. Hepatol..

[72] Kersten V., Seitz T., Sommer J., Thasler W.E., Bosserhoff A., Hellerbrand C. (2023). Bone Morphogenetic Protein 13 Has Protumorigenic Effects on Hepatocellular Carcinoma Cells In Vitro. Int. J. Mol. Sci..

[73] Gaither K.A., Yue G., Singh D.K., Trudeau J., Ponraj K., Davydova N.Y., Lazarus P., Davydov D.R., Prasad B. (2025). Effects of Chronic Alcohol Intake on the Composition of the Ensemble of Drug-Metabolizing Enzymes and Transporters in the Human Liver. J. Xenobiotics.

[74] Zhu S., Chen W., Masson A., Li Y.-P. (2024). Cell signaling and transcriptional regulation of osteoblast lineage commitment, differentiation, bone formation, and homeostasis. Cell Discov..

[75] Wu D.H., Hatzopoulos A.K. (2019). Bone morphogenetic protein signaling in inflammation. Exp. Biol. Med..

[76] Ma N., Teng X., Zheng Q., Chen P. (2019). The regulatory mechanism of p38/MAPK in the chondrogenic differentiation from bone marrow mesenchymal stem cells. J. Orthop. Surg. Res..

[77] Li Q., Gao Z., Chen Y., Guan M.X. (2017). The role of mitochondria in osteogenic, adipogenic and chondrogenic differentiation of mesenchymal stem cells. Protein Cell.

[78] Thoudam T., Gao H., Jiang Y., Huda N., Yang Z., Ma J., Liangpunsakul S. (2024). Mitochondrial quality control in alcohol-associated liver disease. Hepatol. Commun..

[79] Abdelmegeed M.A., Ha S.K., Choi Y., Akbar M., Song B.J. (2017). Role of CYP2E1 in Mitochondrial Dysfunction and Hepatic Injury by Alcohol and Non-Alcoholic Substances. Curr. Mol. Pharmacol..

[80] Gilbert L., He X., Farmer P., Boden S., Kozlowski M., Rubin J., Nanes M.S. (2000). Inhibition of osteoblast differentiation by tumor necrosis factor-alpha. Endocrinology.

[81] Jiang N., Li Y., Shu T., Wang J. (2019). Cytokines and inflammation in adipogenesis: An updated review. Front. Med..

[82] Li M., Yin H., Yan Z., Li H., Wu J., Wang Y., Wei F., Tian G., Ning C., Li H. (2022). The immune microenvironment in cartilage injury and repair. Acta Biomater..

[83] Zhou G., Zheng Q., Engin F., Munivez E., Chen Y., Sebald E., Krakow D., Lee B. (2006). Dominance of *SOX9* function over *RUNX2* during skeletogenesis. Proc. Natl. Acad. Sci. USA.

[84] Tu K.N., Lie J.D., Wan C.K.V., Cameron M., Austel A.G., Nguyen J.K., Van K., Hyun D. (2018). Osteoporosis: A Review of Treatment Options. Pharm. Ther..

[85] Li S., Hong M., Tan H.Y., Wang N., Feng Y. (2016). Insights into the Role and Interdependence of Oxidative Stress and Inflammation in Liver Diseases. Oxidative Med. Cell. Longev..

