# Supplementary material for: Three-Dimensional Human Liver Micro Organoids and Bone Co-Culture Mimics Alcohol-Induced BMP Dysregulation and Bone Remodeling Defects
Source: Cells. 2026 Feb 1;15(3):274. doi: 10.3390/cells15030274 (PMC12896687; doi:10.3390/cells15030274)
Supplement: Supplementary file 1 [file cells-15-00274-s001.zip › cells-4052635-supplementary.pdf]

Supplementary Table S1. List of different medium

Medium	Composition
HepaRG Culture medium	William's Medium E (W1878, Sigma), 10% fetal calf serum (FCS; 10270-106, Life Technologies), 5 μg/mL insulin (Novo Nordisk, Bagsvaerd, Denmark), 2 mM glutamine (GLU; M11-006, Sigma), 50 μM hydrocortisone (Pfizer, New York, USA), and 1% Penicillin/Streptomycin (P/S; P0781, Sigma)
LX-2 Culture medium	Dulbecco's modified Eagle's medium (D5796, Sigma), 2% FCS, 1% P/S, and 1% GLU
Huvec Culture medium	Endothelial Cell Growth Basal Medium 2 (PromoCell, Heidelberg, Germany), 2% FCS, 1% Antibiotic/Antimycotic (Merck, Darmstadt, Germany), 0.5 ng/mL human Vascular Endothelial Growth Factor 165 (PromoCell, Heidelberg, Germany), 10 ng/mL human fibroblast growth factor (PromoCell, Heidelberg, Germany), 5 ng/mL human Epidermal growth factor (PromoCell, Heidelberg, Germany), 20 ng/mL recombinant human insulin-like growth factor (PromoCell, Heidelberg, Germany), 22.5 μg/mL Heparin (Leo Pharma, Bellerup, Denmark), 0.2 μg/mL Hydrocortisone, and 1 μg/mL L-ascorbic acid (Merck, Darmstadt, Germany).
THP-1 Culture medium	RPMI 1640 Medium (R8758, Sigma), 5% FCS
SCP-1 Culture medium	Minimum Essential Medium Eagle Alpha (AL081A, Omni Life Science), 5% FCS
Bone differentiation medium	1% FCS, 200 μM L-ascorbate-2-phosphate (A8960-5G, Sigma), 5 mM β-glycerophosphate (A2253, AppliChem), 25 mM HEPES (HN78.2, Carl Roth), 1.5 mM $CaCl_2$ (CN93.2, Carl Roth), and 20 ng/ml Vitamin D3 (95230, Sigma)
Liver micro-organoids culture medium	50:50 HepaRG culture medium with 1.7% dimethyl sulfoxide (DMSO): Huvec Culture medium
Liver-bone co-culture medium	Liver micro-organoids culture medium, 200 μM L-ascorbate-2-phosphate, 5 mM β-glycerophosphate, 25 mM HEPES, 1.5 mM $CaCl_2$, and 20 ng/ml Vitamin D3

Supplementary Table S2. Human primer sequences used in PCR.

Gene	Accession Number	Forward Primer (5′–3′)	Reverse Primer (3′–5′)	Product Length (bp)	Annealing Temperature (◦C)
Fapα	NM_004460.5	CATCTGGAAAAATGAAGACTTGGGT	CCGATCAGGTGATAAGCCGT	338	60
CYP2E1	NM_000773.3	GACTGTGGCCGACCTGTT	ACTACGACTGTGCCCTTGG	296	59
SNAIL1	NM_005985.3	ACCACTATGCCGCGCTCTT	GGTCGTAGGGCTGCTGGAA	115	62
SNAIL2	NM_003068.5	ACAGCGAACTGGACACACAT	GAGAGAGGCCATTGGGTAGC	168	60
Runx2	NM_001024630.4	CTGTGGTTACTGTCATGGCG	GGGAGGATTTGTGAAGACGGT	170	60
PPARγ	NM_138712.5	TCGAGGACACCGGAGAGGG	AAGTTGGTGGGCCAGAATGG	162	62
SOX9	NM_000346.3	GAAGGACCACCCGGATTACA	GCCTTGAAGATGGCGTTGG	120	60
18S	NR_003286	GGACAGGATTGACAGATTGAT	AGTCTCGTTCGTTATCGGAAT	111	56

Supplementary Table S3. List of antibodies used for Wwestern blot.

Antibody	Host	Company	Cat. No	Dilution
TGFβ-1	Mouse	Santa Cruz Biotechnology	sc-130348	1:1000
PINP	Rabbit	Abbexa	abx131414	1:1000
TNAP	Goat	Santa Cruz Biotechnology	sc-23430	1:1000
TRAP	Rabbit	Abcam	ab191406	1:1000
NTX	Rabbit	Cloud Clone Corp.	PAA639H401	1:1000
MMP9	Mouse	Santa Cruz Biotechnology	sc-21733	1:1000
BMP2	Mouse	Santa Cruz Biotechnology	sc-137087	1:1000
BMP9	Mouse	Santa Cruz Biotechnology	sc-514211	1:1000
BMP13	Mouse	Santa Cruz Biotechnology	sc-374184	1:1000
CYP2E1	Rabbit	Santa Cruz Biotechnology	sc-133491	1:200
Phospho-Smad 1/5	Rabbit	Cell Signaling	9516	1:1000
Phospho-p38	Rabbit	Cell Signaling	4511	1:1000
HPRT	Mouse	Santa Cruz Biotechnology	sc-376938	1:1000
Anti-rabbit IgG	Mouse	Santa Cruz Biotechnology	sc-2357	1:10000
Anti-Mouse IgG	Horse	Cell Signaling	7076	1:10000
Anti-Goat IgG	Donkey	Santa Cruz Biotechnology	sc-2020	1:10000

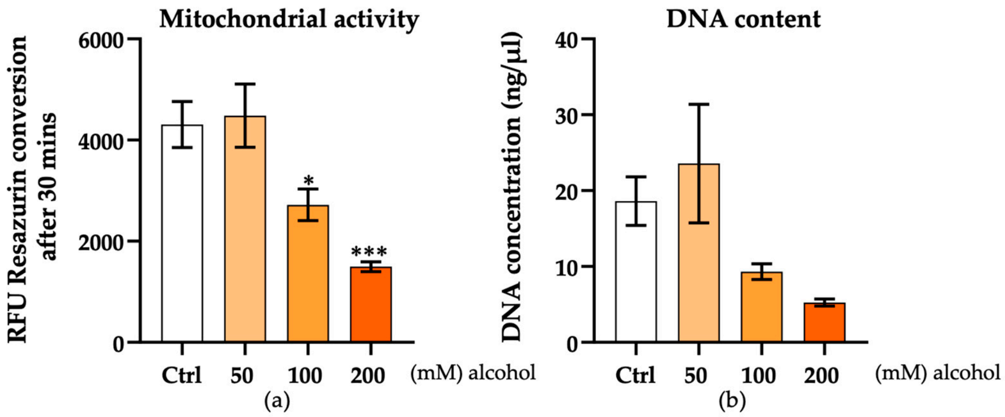

Figure S1. Alcohol toxicity test in 3D liver micro-organoids on day 7. Mitochondrial activity (a) and total DNA content (b) were used to evaluate the effect of alcohol on cell viability. The Kruskal–Wallis test followed by Dunn's multiple comparison test was used to determine statistical differences. Data are presented as means ± SEM, and the significance is shown as * $p < 0.05$, *** $p < 0.001$ vs. the control group. N = 3, n = 3.

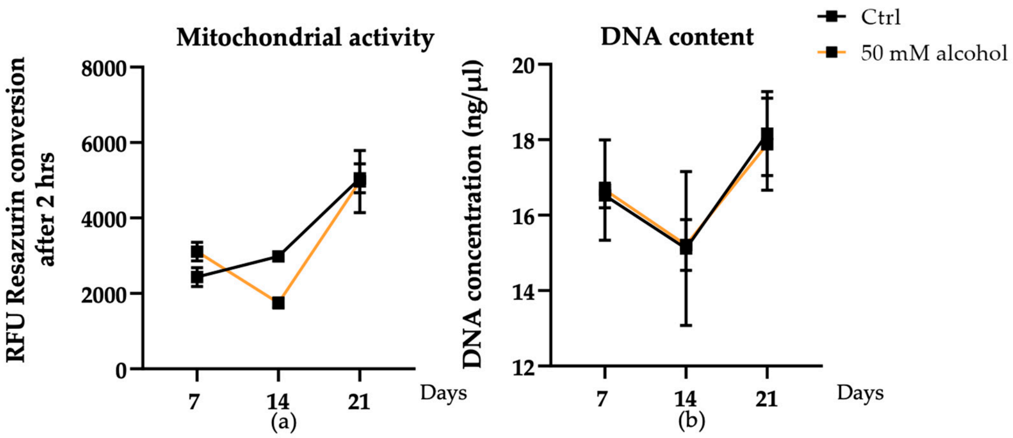

Figure S2. Alcohol toxicity test in the 3D bone co-culture system. Mitochondrial activity (a) and total DNA content (b) were used to evaluate the effect of alcohol on metabolic activity. Data are presented as means ± SEM. N = 3, n = 3.

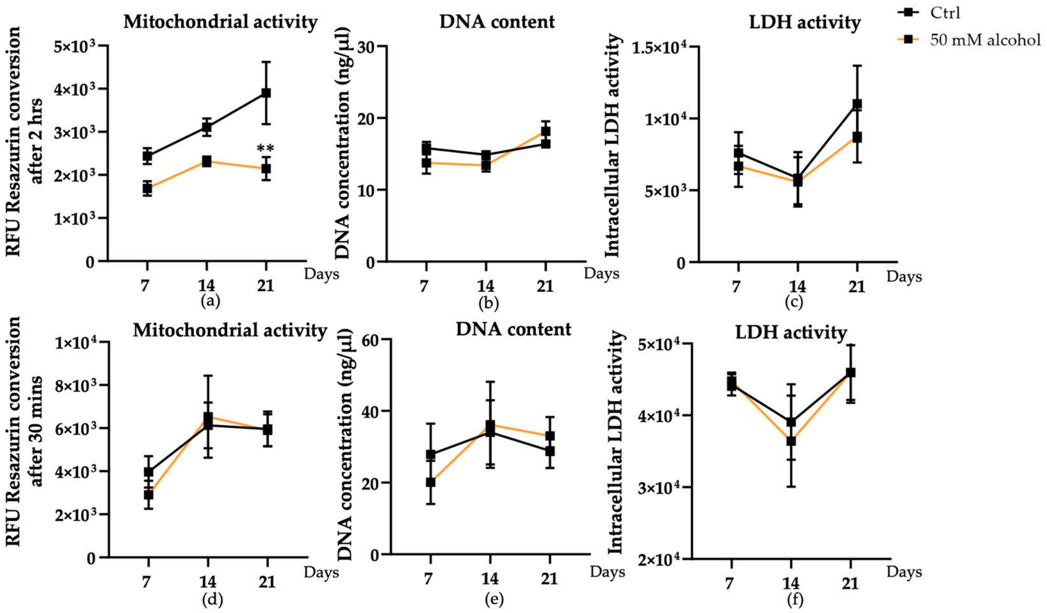

Figure S3. Viability of bone and liver micro-organoids in the liver–bone co-culture system. The viability was assessed on days 7, 14, and 21 using resazurin conversion, DNA concentration, and LDH release. (a), (b), and (c) represented bone-related characteristics, while (d), (e), and (f) showed the cell viability of liver micro-organoids. At each time point, an indirect LDH assay was performed by collecting all cells, applying lysis, and measuring total LDH content to estimate the total number of viable cells in each group. The two-way ANOVA followed by Tukey's multiple comparisons test was used to determine statistical differences. Data are presented as means ± SEM, and the significance is shown as ** $p < 0.01$ vs. the control group. N = 3, n = 3.
